# Bioactive Metabolites from Aerial Parts of *Plantago indica* L.: Structural Elucidation and Integrated In Vitro/In Vivo Assessment of Anti-Inflammatory and Wound-Healing Efficacy

**DOI:** 10.3390/plants15010141

**Published:** 2026-01-03

**Authors:** Hilal Bacanak, Zeynep Dogan, Esra Küpeli Akkol, Akito Nagatsu, Iclal Saracoglu

**Affiliations:** 1 Department of Pharmacognosy, Faculty of Pharmacy, Hacettepe University, Ankara 06100, Türkiye; zeynep.ocak@hacettepe.edu.tr (Z.D.); isaracog@hacettepe.edu.tr (I.S.); 2 Department of Pharmacognosy, Faculty of Pharmacy, Tokat Gaziosmanpaşa University, Tokat 60100, Türkiye; 3 Department of Pharmacognosy, College of Pharmacy, Kinjo Gakuin University, Nagoya 463-8521, Japan; anagatsu@kinjo-u.ac.jp; 4 Department of Pharmacognosy, Faculty of Pharmacy, Gazi University, Ankara 06330, Türkiye; 5 Department of Pharmacognosy, Faculty of Pharmacy, Lokman Hekim University, Ankara 06510, Türkiye

**Keywords:** biological activity, anti-inflammatory, wound healing, phytochemistry, *Plantago indica*, Plantaginaceae

## Abstract

The genus *Plantago* (Plantaginaceae) is widely distributed worldwide. The *Plantago* species are used in traditional medicine as wound healers, anti-inflammatory agents, antipyretics, and analgesics. This study aimed to investigate the phytochemical composition from the aerial parts of *Plantago indica* L. and to evaluate its biological activities. Isolation studies and in vitro investigations were conducted on an aqueous phase of 80% EtOH extract of *Plantago indica*. In addition, in vivo studies were carried out using the MeOH, 80% EtOH, and water extracts. Plantarenaloside (**1**), 3-oxo-*α*-ionol *β*-glucoside (**2**), martynoside (**3**), acteoside (**4**), feruloyl gardoside (**5**), and ursolic acid (**6**) were isolated from the extract. The structures of the compounds were elucidated using 1D- and 2D-NMR and ESI-MS analyses. The extract, fractions, and pure compounds were tested in vitro for cytotoxicity (MTT), anti-inflammatory activity (NO, IL-6, and TNF-*α* production), wound healing (scratch test), and antioxidant capacity (DPPH, ABTS, SO). Feruloyl gardoside (20.11–58.27%) significantly reduced NO levels at concentrations of 25–100 µM. It significantly reduced IL-6 levels (40.17%) at 100 µM. Additionally, the in vivo anti-inflammatory (acetic acid-induced vascular permeability) and wound healing (incision and excision models) effects of the extracts were investigated. The findings suggest that *P. indica* may be considered to be a potential therapeutic option for managing inflammation and for promoting wound healing.

## 1. Introduction

The genus *Plantago*, belonging to the Plantaginaceae family, includes 246 species distributed globally [1]. In Türkiye, there are a total of 23 species, among which the endemic species *P. anatolica* and *P. euphratica* are also included [2].

Various *Plantago* species are commonly utilized in traditional medicine due to their antimicrobial, antidiabetic, antispasmodic, antiviral, anti-inflammatory, wound healing, and diuretic properties [3,4]. Ethnobotanical research in Türkiye has demonstrated the extensive use of these plants in folk medicine for the treatment of various conditions, such as abdominal pain, abscesses, wounds, burns, diabetes, cough, bronchitis, constipation, hemorrhoids, sore throat, and rheumatism [5,6,7]. Specifically, in traditional Turkish medicine, the seeds of *P. indica* are favored for their laxative properties [8]. On a global scale, the seeds are also recognized for their ability to stimulate blood circulation due to their high soluble fiber content [9]. In addition, it has been reported that the *Plantago* species are consumed as food by adding them to salads, soups, cakes, and bread, and are used in the preparation of tea, fruit juice, wine, cereals, and ice cream [10].

Phytochemical analyses have indicated that the *Plantago* species contain diverse secondary metabolites, including iridoids, phenylethanoid glycosides, flavonoids, tannins, triterpenes, saponins, and sterols [4,11]. Aucubin and catalpol in the iridoid structure, and acteoside and plantamajoside in the phenylethanoid glycoside structure are very important for the chemotaxonomic identification of the *Plantago* species [9].

The pharmacological activities of the *Plantago* species have been supported by numerous in vitro and in vivo studies, confirming their antioxidant, antimicrobial, cytotoxic, antidiarrheal, immunomodulatory, and antihypertensive effects [12,13,14,15,16,17]. Many studies have also highlighted the significant role of the *Plantago* species in anti-inflammatory and wound healing effects [18,19,20,21,22,23,24].

Studies specifically focusing on *P. indica* have been relatively limited. One study analyzed 14 iridoid compounds across 14 different *Plantago* species, revealing their taxonomic relationships and identifying plantarenaloside and aucubin in *P. indica*, which belongs to the Psyllium subgenus [25]. In another study, lipid and fatty acid profiles in the seeds from six plants, including *P. indica*, were evaluated. This study concluded that *P. indica* seeds have a low total oil content (4.36 g/100 g) but a high γ-linolenic acid content (13.13% of unsaturated fatty acids) [26].

The anti-inflammatory and wound healing properties of the genus *Plantago* are well-documented in the literature. In our previous studies, we conducted comprehensive investigations on these biological activities in various *Plantago* species [19,27]. In this context, the present study is a continuation of our previous studies. Wound healing is a multifactorial process involving ROS species, inflammatory response, and tissue regeneration [28,29]. Therefore, the aim of this study was to investigate the antioxidant, anti-inflammatory, and wound-healing activities together. For this purpose, the phytochemical characterization from an aqueous phase of the 80% EtOH extract of the plant was contributed and its antioxidant activity was evaluated using 2,2-diphenyl-1-picrylhydrazyl (DPPH), 2,2′-azino-bis(3-ethylbenzothiazoline-6-sulfonic acid) (ABTS), and superoxide (SO) radical scavenging assays. To determine the anti-inflammatory effect, the nitric oxide (NO), interleukin-6 (IL-6), and tumor necrosis factor alpha (TNF-*α*) levels were measured in lipopolysaccharide (LPS)-stimulated RAW 264.7 macrophage cells in vitro and an acetic acid-induced vascular permeability test was performed in vivo. The wound healing potential was investigated using an in vitro scratch assay and in vivo linear incision and circular excision wound models.

## 2. Results

### 2.1. Characterization of the Isolated Compounds

**Figure 1:**
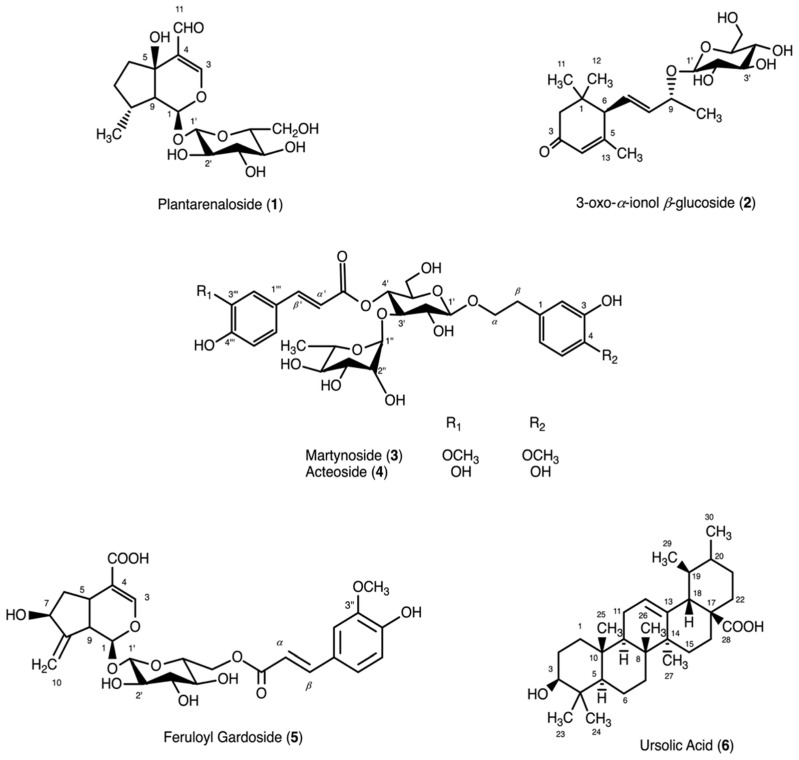
Chemical structures of compounds isolated from *Plantago indica*.

The method for preparing the 80% EtOH extract from the aerial parts of *P. indica* is described in detail in Section 4.3. The water-soluble fraction of the 80% EtOH extract was subjected to various chromatographic methods. As a result, six compounds were isolated. The structures of the isolated compounds were determined by comparing 1D- and 2D-NMR (^1^H, ^13^C, HMQC, HMBC) and ESI-MS data (Supplementary Files) with data from the literature. The compounds were identified as plantarenaloside (**1**), 3-oxo-*α*-ionol *β*-glucoside (**2**), martynoside (**3**), acteoside (**4**), feruloyl gardoside (**5**), and ursolic acid (**6**) [19,30,31,32,33,34]. The chemical structures of all compounds isolated from *P. indica* are presented in Figure 1.

### 2.2. In Vivo Biological Activities

#### 2.2.1. Acetic Acid-Induced Capillary Permeability Results

The results of the present study revealed that the MeOH and 80% EtOH extracts prepared from the aerial parts of *P. indica* exhibited remarkable anti-inflammatory activity. As presented in Table 1, the administration of 80% EtOH extract at a dose of 100 mg/kg to mice resulted in 35.3% inhibition, while the MeOH extract at a dose of 100 mg/kg caused 26.6% inhibition in the acetic acid-induced capillary permeability test.

#### 2.2.2. Linear Incision Wound Model

The resistance of the repaired tissue to breaking under tension is referred to as tensile strength. Tensile strength is determined in the linear incision wound model. The repaired tissue is removed and its tensile strength is measured [35]. It was concluded that the 80% EtOH extract of *P. indica* promoted wound healing in rats at a statistically significant level, with a value of 26.5% (Table 2).

#### 2.2.3. Circular Excision Wound Model

A circular excision wound model was used to measure the percentage reduction in the wound area [36]. In mice, the 80% EtOH extract demonstrated statistically significant wound healing on days 6, 8, 10, and 12, with a wound contraction rate of 37.7% on day 12. The MeOH extract also showed a significant wound healing effect on days 6, 8, and 10, reaching 33.7% wound contraction on day 10 (Table 3).

### 2.3. In Vitro Biological Activities

#### 2.3.1. Cell Viability Assay (MTT)

**Figure 2:**
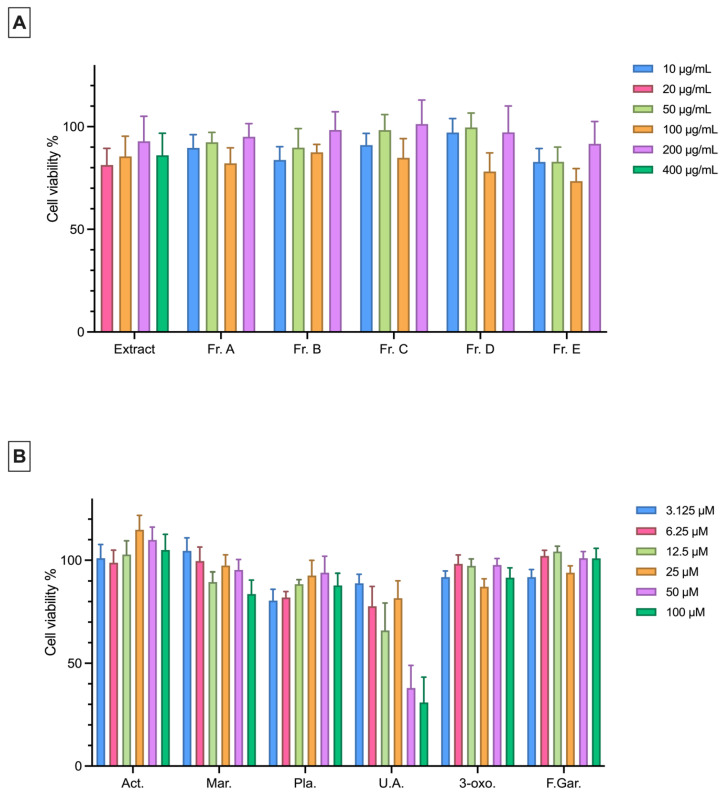
Cytotoxic effects of the extract, fractions (**A**) and pure compounds (**B**) at different concentrations on L929 cells (10^5^ cells/mL) as determined by the MTT assay. Data are presented as the mean ± SEM from three independent experiments. Act: Acteoside; Mar: Martynoside; Pla: Plantarenaloside; U.A.: Ursolic Acid; 3-oxo: 3-oxo-*α*-ionol *β*-glucoside; F.Gar.: Feruloyl Gardoside.

**Figure 3:**
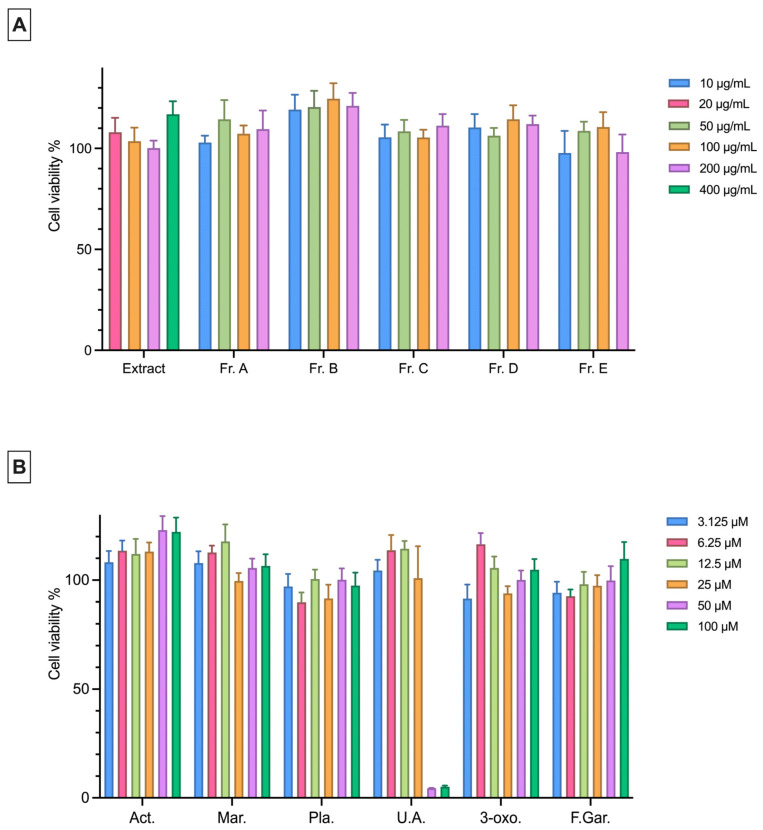
Cytotoxic effects of the extract, fractions (**A**) and pure compounds (**B**) at different concentrations on RAW 264.7 cells (5 × 10^5^ cells/mL) as determined by the MTT assay. Data are presented as the mean ± SEM from three independent experiments. Act: Acteoside; Mar: Martynoside; Pla: Plantarenaloside; U.A.: Ursolic Acid; 3-oxo: 3-oxo-*α*-ionol *β*-glucoside; F.Gar.: Feruloyl Gardoside.

An MTT assay was performed to determine the non-cytotoxic concentrations of the extract, fractions, and isolated pure compounds on the L929 and RAW 264.7 cell lines. In both cell lines, the extract was applied at concentrations of 20, 100, 200, and 400 μg/mL, while the fractions were tested at 10, 50, 100, and 200 μg/mL (Figure 2A and Figure 3A). The pure compounds were prepared in six different concentrations ranging from 100 to 3.125 μM using a two-fold serial dilution. It was concluded that the extract and fractions did not exhibit any cytotoxic effect on both cell lines at the tested concentrations. For pure compounds, cell viability in the L929 and RAW 264.7 cell lines was found to be above 80% at almost all concentrations except for ursolic acid at 50 and 100 μM (Figure 2B and Figure 3B).

#### 2.3.2. Effects on LPS-Induced NO, IL-6, and TNF-α Cytokine Production

**Figure 4:**
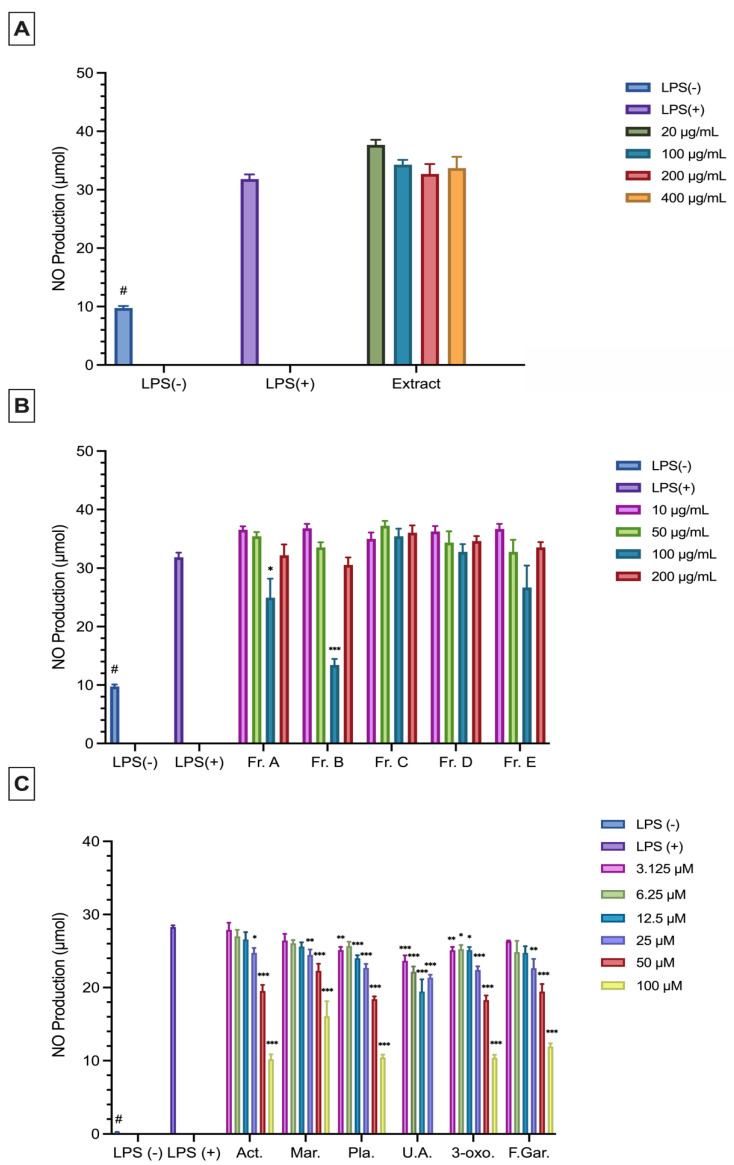
Anti-inflammatory effects of the extract (**A**), fractions (**B**), and pure compounds (**C**) at different concentrations on RAW 264.7 cells (5 × 10^5^ cells/mL) as determined by NO production. Data are presented as the mean ± SEM from three independent experiments. LPS (+), positive control; LPS (−), negative control. # *p* < 0.05 compared to the LPS (−) group; *: *p* < 0.05; **: *p* < 0.01; ***: *p* < 0.001. The sample materials and the reference material were compared to LPS (+). Act: Acteoside; Mar: Martynoside; Pla: Plantarenaloside; U.A.: Ursolic Acid; 3-oxo: 3-oxo-*α*-ionol *β*-glucoside; F.Gar.: Feruloyl Gardoside.

**Figure 5:**
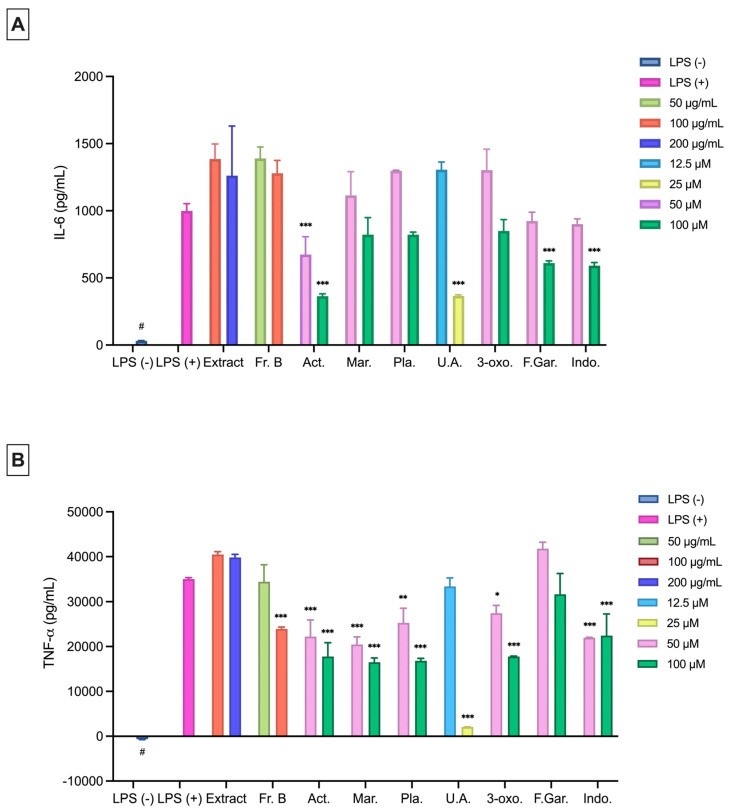
Anti-inflammatory effects of the extract, fractions, and pure compounds at different concentrations on RAW 264.7 cells (5 × 10^5^ cells/mL) as determined by IL-6 (**A**) and TNF-*α* (**B**) production. Data are presented as the mean ± SEM from three independent experiments. LPS (+), positive control; LPS (−), negative control. # *p* < 0.05 compared to the LPS (−) group; *: *p* < 0.05; **: *p* < 0.01; ***: *p* < 0.001. The sample materials and the reference material were compared to LPS (+). Act: Acteoside; Mar: Martynoside; Pla: Plantarenaloside; U.A.: Ursolic Acid; 3-oxo: 3-oxo-*α*-ionol *β*-glucoside; F.Gar.: Feruloyl Gardoside; Indo: Indomethacin.

The extract did not reduce the levels of NO, IL-6, and TNF-*α* significantly in LPS-stimulated RAW 264.7 macrophages compared to the LPS (+) group (Figure 4 and Figure 5). Among the fractions, Fr. A (31.14% inhibition) and Fr. B (83.31%) significantly reduced NO production at a concentration of 100 μg/mL compared to the LPS (+) group (Figure 4B). Fr. B (23.93%) was found to significantly reduce the TNF-*α* levels at a concentration of 100 μg/mL (Figure 5B). Ursolic acid (16.47–31.51%) and 3-oxo-*α*-ionol *β*-glucoside (10.77–63.71%) significantly inhibited NO production at all the tested concentrations (Figure 4C). All other isolated compounds significantly reduced NO production at 25, 50, and 100 μM (13.62–64.45%) (Figure 4C). Additionally, plantarenaloside significantly inhibited NO production even at 3.125 µM, with an inhibition rate of 11.27% (Figure 4C).

Acteoside significantly inhibited IL-6 cytokine production at 50 and 100 μM (33.57–65.66%) concentrations, while feruloyl gardoside (40.17%) and indomethacin (42.12%) showed a similar inhibition at 100 μM (Figure 5A). Ursolic acid (65.42%) significantly inhibited IL-6 production at 25 μM (Figure 5A). All compounds, except for feruloyl gardoside, significantly reduced the TNF-*α* levels at 50 and 100 μM (21.37–52.00%) (Figure 5B). Additionally, ursolic acid significantly inhibited TNF-*α* production at 25 μM concentrations (Figure 5B).

#### 2.3.3. Scratch Assay Results

The wound healing effects of the extract, fractions, and isolated pure compounds on the L929 cell line were evaluated using the scratch assay method. The wound area was measured at 0 and 24 h. The extract was tested at concentrations of 20, 100, 200, and 400 μg/mL, while the fractions were tested at 10, 50, 100, and 200 μg/mL. The pure compounds were examined at six different concentrations ranging from 100 to 3.125 μM through two-fold serial dilution. The in vitro wound healing effect of the samples were evaluated statistically by comparing them with the FBS (−) control group. Among the fractions, Fr. B showed the highest effect at a concentration of 200 μg/mL (Closure %: 90.83%; *p* < 0.001). Fr. B, C, and D showed significant effects at almost all concentrations (Table 4).

Plantarenaloside, martynoside, acteoside, and feruloyl gardoside were found to be significantly effective at all concentrations. Among the pure compounds, 3-oxo-*α*-ionol *β*-glucoside exhibited the highest wound healing activity at a concentration of 12.5 μM (Closure %: 75.2%; *p* < 0.001) (Table 5).

#### 2.3.4. Antioxidant Activity

The *P. indica* extract and the fractions were evaluated for their scavenging activities against the DPPH, ABTS, and SO radicals at concentrations of 50, 100, 200, and 400 µg/mL for the extract and at concentrations of 25, 50, 100, and 200 μg/mL for the fractions. The extracts and fractions showed a radical scavenging potential on the DPPH, ABTS, and SO radicals, with an increasing effect as the concentration increased. Quercetin, a natural compound known for its antioxidant activity, was used as the reference compound in the experiments.

**Figure 6:**
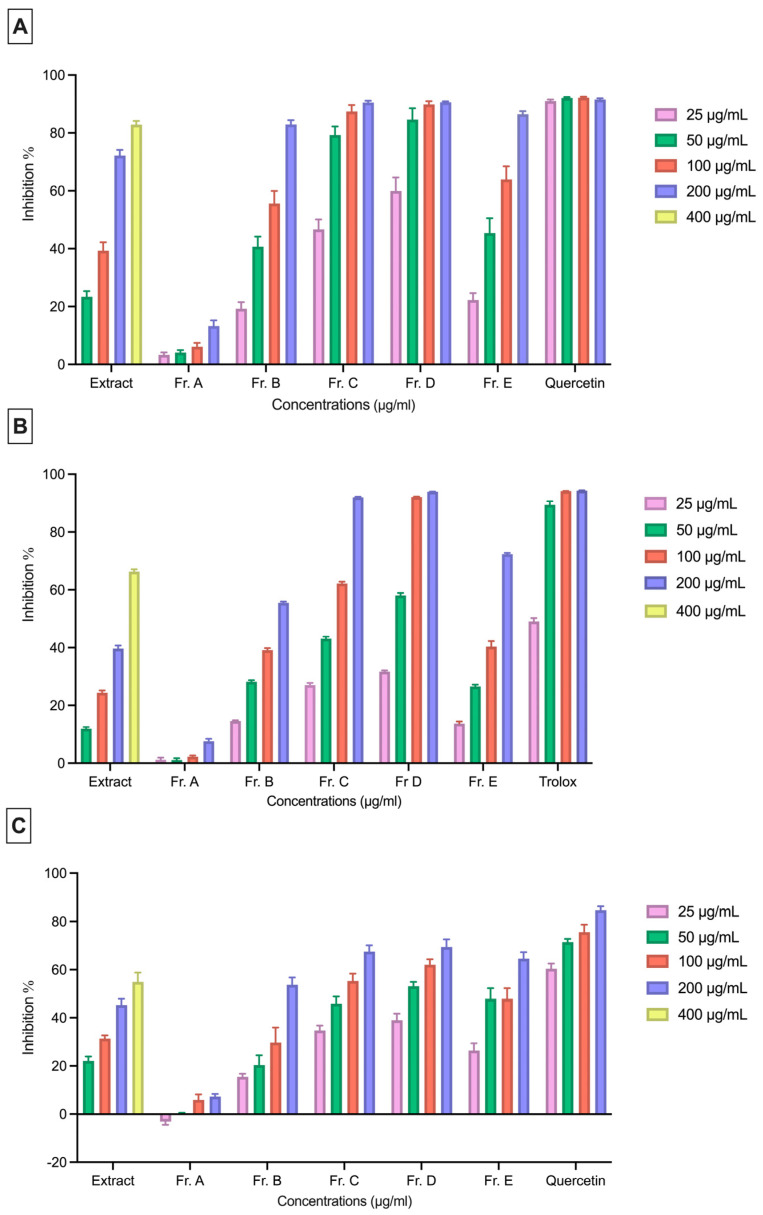
DPPH (**A**), ABTS (**B**), and SO (**C**) radical scavenging activities of fractions of *P. indica* and the reference compound. Data are expressed as the mean ± SEM of three independent experiments.

The inhibitory concentration 50% (IC_50_) value of the extract against the DPPH radical was measured as 160.62 μg/mL. Among the fractions, Fr. C (IC_50_: 15.13 μg/mL) and Fr. D (IC_50_: 12.93 μg/mL) exhibited a higher DPPH radical scavenging activity compared to the other fractions (Figure 6A).

The extract showed an IC_50_ value of 283.40 μg/mL against the ABTS radical. Among the fractions, Fr. C (IC_50_: 76.69 μg/mL) and Fr. D (IC_50_: 44.85 μg/mL) demonstrated a notably stronger ABTS radical scavenging activity than the other fractions (Figure 6B).

The extract exhibited an IC_50_ value of 228.80 μg/mL against the SO radical. Similar to the results observed with the DPPH and ABTS radical scavenging activities, Fr. C (88.82 μg/mL) and Fr. D (55.24 μg/mL) showed the strongest SO radical scavenging effects (Figure 6C).

## 3. Discussion

The antidiabetic, antimicrobial, and cytotoxic effects of the *Plantago* species, as well as their anti-inflammatory and wound healing activities, have been demonstrated using various in vitro and in vivo experimental models [14,19,37,38]. In the present investigation, phytochemical studies were performed on the aerial parts of *P. indica* and its anti-inflammatory and wound healing effects were demonstrated through both in vitro and in vivo experiments.

In our research, fractionation was performed considering the chemical properties of the major secondary metabolites found in the *Plantago* species. An aqueous phase of 80% EtOH extract was fractionated using a polyamide column. This allowed for the separation of phenolic and terpenic compounds according to their polarity. Fractions were selected based on their TLC profiles and biological activities were investigated according to this chemical content.

Wound healing is a multifaceted process involving anti-inflammatory and radical-scavenging activities [39]. Because healing, inflammation, and oxidative stress are closely interconnected, the combined evaluation of these three processes was deemed to be important and included in our study. Reactive oxygen species (ROS) are important signaling molecules that play a role in the early stages of wound healing, but excessive ROS production can negatively affect the healing process. Therefore, a close relationship between antioxidant activity and wound healing is known. In this study, the results of the DPPH, ABTS, and SO radical scavenging tests were evaluated together with the in vitro scratch test findings. The results show that fractions with high antioxidant capacity (Fr. C, Fr. D, and Fr. E) exhibit more pronounced wound-healing activity. Previous studies have shown that the *Plantago* species possess significant antioxidant activity. Research on the extracts obtained from different *Plantago* species and plant parts has reported significant free radical scavenging effects using various in vitro methods, such as DPPH, ABTS, and NO [12,40,41]. Consistent with the data reported in the literature, the present study also demonstrates that *P. indica* possesses antioxidant activity using different in vitro methods.

Among the obtained compounds, plantarenaloside (**1**) and feruloyl gardoside (**5**) are iridoid glycosides, while 3-oxo-*α*-ionol *β*-glucoside (**2**) is a megastigmane glycoside, martynoside (**3**) and acteoside (**4**) are phenylethanoid glycosides, and ursolic acid (**6**) is a triterpene [19,30,31,32,33,34]. The predominance of iridoid and phenylethanoid glycosides among the isolated compounds is consistent with the literature reporting that these groups of compounds are characteristic of the *Plantago* species. Furthermore, upon review of the literature, it was understood that 3-oxo-*α*-ionol *β*-glucoside (**2**) and feruloyl gardoside (**5**) were isolated from a *Plantago* species for the first time with this study.

The anti-inflammatory and wound healing effects of three different extracts were also evaluated using in vivo models. In the acetic acid-induced capillary permeability assay, both the MeOH and 80% EtOH extracts exhibited significant activity. In the linear incision wound model, only the 80% EtOH extract was found to be effective, while in the circular excision model, both the MeOH and 80% EtOH extracts demonstrated notable wound healing activity. By contrast, the aqueous extract showed minimal anti-inflammatory and wound healing effects. Considering the TLC profiles of *P. indica*, it was concluded that the alcoholic extracts are richer in secondary metabolites compared to the aqueous extract. These findings for *P. indica* are consistent with other in vivo studies in the literature demonstrating the wound healing and anti-inflammatory effects of the *Plantago* species [42,43,44].

The cytotoxic effects of the extract, its fractions, and its isolated compounds were tested on the L929 and RAW 264.7 cell lines. Except for ursolic acid, no cytotoxic effects were observed on either cell line. Ursolic acid exhibited cytotoxicity at 50 and 100 μM concentrations in both cell lines, consistent with previous reports [45].

In LPS-stimulated RAW 264.7 cells, the levels of NO, IL-6, and TNF-*α* were measured. Fr. A and Fr. B significantly reduced the NO levels. Previous studies have demonstrated the anti-inflammatory activities of iridoids [46]. Considering the TLC profiles and phytochemical analysis results, Fr. A and Fr. B fractions were determined to be rich in iridoid compounds, and it is thought that the observed anti-inflammatory effects may be related to this group of compounds. Similarly, plantarenaloside significantly reduced the NO and TNF-*α* levels at nearly all concentrations tested, likely due to its classification as an iridoid glycoside. In a study conducted on *Penstemon gentianoides*, a species belonging to the Plantaginaceae family, plantarenaloside was isolated and its effect on nitric oxide (NO) production was evaluated in LPS-stimulated RAW 264.7 macrophage cells. Plantarenaloside was studied at a concentration of 25 and 50 μg/mL. The results demonstrated that plantarenaloside inhibited NO production, which may be attributed to the suppression of iNOS enzyme activity or the down regulation of its gene expression. These findings support the anti-inflammatory potential of plantarenaloside [47]. In our present study, plantarenaloside significantly reduced NO levels (11.27–63.57%) in LPS-stimulated RAW 264.7 cells at all concentrations tested except 6.25 μM. These findings are consistent with the NO production inhibitory effect of plantarenaloside reported in the literature.

Ursolic acid significantly reduced the levels of NO, IL-6, and TNF-*α* at non-cytotoxic concentrations. A previous study showed that ursolic acid reduced the release of pro-inflammatory mediators, such as TNF-*α*, IL-6, and IL-1*β*, in LPS-stimulated macrophage cells and that this effect was associated with the inhibition of the TLR4/MyD88 signaling pathway. Furthermore, ursolic acid has been reported to modulate the inflammatory response by inducing autophagy in macrophages [48]. In another study, the effect of ursolic acid at 5- and 10-μM concentrations on reducing the NO, IL-6, and TNF-*α* levels in LPS-stimulated RAW 264.7 cells were investigated. It was concluded that ursolic acid significantly reduced the NO and IL-6 levels at both concentrations and the TNF-*α* level at 10 μM concentration [49]. In our study, ursolic acid (16.47–31.51%) significantly inhibited the NO levels in LPS-stimulated RAW 264.7 cells at all concentrations tested (3.125–25 μM). It also significantly reduced the IL-6 and TNF-*α* levels at 25 μM. These findings are consistent with the results reported in the literature.

The compound 3-oxo-*α*-ionol *β*-glucoside was found to exert a suppressive effect on inflammatory mediators, such as NO, IL-6, and TNF-*α*, in LPS-stimulated RAW 264.7 cells. In one study, the effect of 3-oxo-*α*-ionol *β*-glucoside on NO production in LPS-stimulated RAW 264.7 cells were investigated. The compound was tested at concentrations of 12, 25, 50, and 100 μM, and it was found to significantly inhibit NO production at 100 and 50 μM concentrations [50]. In our current study, 3-oxo-*α*-ionol *β*-glucoside significantly reduced NO production (10.77–63.71%) at all doses in the concentration range of 3.125–100 µM, and the obtained data are consistent with the existing literature.

Acteoside and martynoside significantly reduced the levels of NO with the ratio of 12.58–64.47% and 13.63–43.50% at the concentration of 25–100 μM, respectively. Additionally, these compounds decreased the production of TNF-*α* by 35.95–48.39% and 40.91–52.00% at the concentration of 50–100 μM, respectively. In addition, acteoside significantly inhibited IL-6 levels by 33.57 to 65.66% at concentrations of 50–100 μM, respectively. In our previous study investigating the anti-inflammatory effect of acteoside, the compound was tested at concentrations of 1, 10, 25, 50, and 100 μg/mL to evaluate its effect on NO production in LPS-stimulated RAW 264.7 cells. Significant inhibition of NO production was observed at 1 and 50 μg/mL concentrations. Additionally, the effects of the compound on the PGE_2_ and TNF-*α* levels were investigated at concentrations of 10, 50, and 100 μg/mL, and it was found to be effective at all tested concentrations. When evaluated together with our current study, it was concluded that the anti-inflammatory effects of these compounds may be associated with their phenolic structures [19].

In the scratch assay, Fr. B (47.07–90.83% wound closure) showed a greater effect compared to the other fractions. This was attributed to the high phenolic content of Fr. B. Among the isolated compounds, 3-oxo-*α*-ionol *β*-glucoside (30.85–75.20% wound closure) exhibited a more pronounced wound healing effect than the others. Additionally, martynoside (14.71–66.13% wound closure) also demonstrated a strong effect. In one study, water and EtOH extracts were prepared separately from the dried leaves of *P. major* and a combined extract was obtained by mixing these two extracts in equal volumes. Additionally, a water extract was prepared from the fresh leaves. Using a scratch assay, the wound healing effects of these four extracts were evaluated on oral epithelial cells at concentrations of 0.1, 1.0, and 10.0 mg/mL. As a result, while all extracts were found to be effective at most of the tested concentrations, the EtOH extract at 10.0 mg/mL showed the highest activity [24]. In another study, a 70% EtOH extract standardized with acteoside was prepared from the leaves of *P. australis*. The wound healing effects of the extract and acteoside were investigated using a scratch assay on HaCaT cells. At a concentration of 25 μg/mL, the extract showed 81.06% wound closure, while acteoside exhibited 58.70–57.77% wound closure at 5 and 10 μg/mL, respectively [20]. In the present study, an 80% EtOH extract prepared from *P. indica* provided significant wound closure of 30.83% at a concentration 100 μg/mL. On the other hand, acteoside showed significant wound closure (19.95–62.58%) at all concentrations in the range of 3.125–100 μM. The results obtained are consistent with the literature for acteoside. The studies investigating the in vitro effects of the *Plantago* species using a scratch assay are in agreement with our current findings. The wound healing activity of the *Plantago* species has been scientifically demonstrated, thereby supporting their traditional use in wound treatment.

The results of in vitro and in vivo biological effect experiments conducted on *P. indica* scientifically support the use of the *Plantago* genus in traditional medicine. Both *P. indica* and its isolated compounds appear to hold therapeutic potential for the treatment of wounds and inflammation.

## 4. Materials and Methods

### 4.1. Plant Material

Aerial parts of *P. indica* L. were collected in July 2021 at the flowering stage from Incesu Beach, Atakum, Samsun, Türkiye. The plant was identified by Assistant Professor Fergan Karaer (Ondokuz Mayıs University, Faculty of Education, Department of Science). A voucher specimen has been deposited in the herbarium of the Faculty of Pharmacy at Hacettepe University (HUEF 21022). The plant material was dried under shade and powdered prior to extraction.

### 4.2. Chemicals, Reagents and Instruments

MeOH (≥99.7%, purity), EtOH (≥99.8%, purity), CHCl_3_ (≥99.8%, purity), and petroleum ether (40–70 °C, analytical grade) were purchased from Sigma-Aldrich (St. Louis, MO, USA). Methanol-d4 (≥99.8%, %D) and Pyridine-d5 (≥99.5%, %D) were obtained from Isotec, Inc. (Miamisburg, OH, USA).

Open column chromatography was carried out using various stationary phases, including silica gel [Merck, Kieselgel 60, 70–230 mesh (Darmstadt, Germany) and Fuji BW-200 (Fuji Silysia Chemical, Kasugai, Japan)], octadecylsilyl silica [Merck, 40–63 μm (Darmstadt, Germany) and Cosmosil 140C_18_-OPN (Nacalai Tesque, Inc., Kyoto, Japan)], polyamide [Fluka, polyamide 6, 50–160 μm (Sigma-Aldrich, St. Louis, MO, USA)], and Sephadex [LH-20 (Pharmacia, Uppsala, Sweden)]. Aluminium (Silica gel 60 F_254_, 0.2 mm, Merck, Darmstadt, Germany) and glass plates were used in thin-layer chromatography (TLC) (Silica gel 60 F_254_, 9.5–11.5 µm, Merck, Darmstadt, Germany). Dulbecco’s Modified Eagle Medium (DMEM), LPS, Fetal bovine serum (FBS), and 3-(4,5-dimethylthiazol-2-yl)-2,5-diphenyltetrazolium bromide (MTT) were purchased from Sigma-Aldrich Chem. Co. (St. Louis, MO, USA). Dulbecco’s phosphate-buffered saline was purchased from Pan-Biotech (Aidenbach, Germany), and penicillin-streptomycin was purchased from Gibco Invitrogen Life Technologies (Waltham, MA, USA).

Compounds were concentrated by vacuum rotary evaporators [OSB-2200 (Eyela, Tokyo Rikakikai Co., Ltd., Tokyo, Japan) and Buchi R-210 (Büchi Labortechnik AG, Flawil, Switzerland)]. Microplate readings were performed using a BioTek μQuant microplate reader (MQX200, Winooski, VT, USA). The HPLC (high-performance liquid chromatography) system was equipped with an EYELA (Tokyo Rikakikai Co., Ltd., Tokyo, Japan) Micro Feeder MP-Σ pump, a UV-Vis detector (Jasco UV-2075 Plus, Jasco Co., Tokyo, Japan), and a Develosil RPAQUEOUS column [5 µm; 20 mm × 250 mm] (Nomura Chemical Co., Ltd., Aichi, Japan). Nuclear magnetic resonance (NMR) spectra were measured using a JEOL JMN-ECA600 (600 MHz) spectrometer (Jeol Ltd., Tokyo, Japan) with JEOL Delta software (version 6.4). Wound closure in the scratch assay was determined using a Leica DM IL LED (Wetzlar, Germany) light microscope and the Leica ICC50 W/E digital camera system integrated into this microscope, with the wound area directly determined by the measurement tools on the microscope screen.

### 4.3. Extraction, Fractionation, and Isolation Process

The starting point of this study was the strong ethnobotanical evidence indicating the traditional use of the *Plantago* species for wound healing and anti-inflammatory purposes. In line with this traditional knowledge, the first aim was to demonstrate the efficacy of *P. indica* through in vivo models. Subsequently, in vitro biological assays and phytochemical analyses were conducted to better understand the observed biological activity at the molecular level. Isolation studies and in vitro experiments were performed using an 80% EtOH extract, while in vivo experiments were conducted using MeOH, 80% EtOH, and aqueous extracts.

Approximately 10 g of powdered plant material was used for the MeOH and 80% EtOH extracts in the in vivo experiments. The extracts were obtained by extracting the plant material with the respective solvents (100 mL; 1:10 *w*/*v* ratio) 3 times at 40 °C for 8 h. The obtained filtrates were combined and the solvent was removed under vacuum to obtain the MeOH (2.26 g) and 80% EtOH (2.53 g) extracts. For the water extract, 6.62 g of plant material was weighed and boiled with 500 mL of water for 30 min, then filtered [8]. The water in the resulting extract was evaporated and removed. Thus, the water extract (1.37 g) was obtained. These three extracts were used in the in vivo experiments. Due to the higher efficacy of the 80% EtOH extract and its similar TLC profile to the MeOH extract, this extract was selected for phytochemical and in vitro studies.

Phytochemical analyses and in vitro biological activity studies were carried out on the 80% EtOH extract. For this purpose, 460.3 g of powdered plant material was extracted five times with 3 L of 80% EtOH at 40 °C for 8 h and filtered. The obtained filtrates were combined and the solvent was removed under vacuum to obtain a total of 89.65 g (19.5% *w*/*w*) of crude extract. The crude extract was dissolved in water and then the lipophilic compounds and chlorophylls were removed using petroleum ether. The obtained aqueous phase (50 g) was fractionated using a polyamide column. Elution was started with 100% water and was carried out with 75:25, 50:50, 25:75 H_2_O/MeOH and 100% MeOH, respectively. In this way, 5 fractions were obtained from the polyamide column (Frs. A-E).

Fr. A (1.51 g) was subjected to silica gel column chromatography (SCC) (150 g) using CHCl_3_/MeOH (100:0 to 75:25) as the solvent system to give Frs. A_1–19_. Fr. A_13_ (150 mg) was applied to a vacuum liquid chromatography column filled with Silicagel-C_18_ (20 g) using H_2_O/MeOH (90:10 to 75:25) and thereby Compound **1** (87.5 mg) was isolated. Fr. A_7_ was applied to HPLC system and the flow rate was 4 mL/min. Compound **2** (6.4 mg) was purified with an H_2_O/MeOH (55:45) solvent system.

Fr. D (1.4 g) was subjected to SCC (150 g) using CHCl_3_/MeOH (100:0 to 75:25) as the solvent system to give Fr. D_1–19_. Fr. D_10_ was applied to the HPLC system at a flow rate of 4 mL/min. Compound **3** (7.3 mg) was purified with the solvent system H_2_O/MeOH (45:55). Fr. D_14_ (150 mg) was applied to Sephadex LH-20 CC (50 g, MeOH) and obtained 6 subfraction, D_14a–e_. D_14d_ was applied to the HPLC system. The flow rate was set at 4 mL/min. Compound **4** (4.5 mg) was obtained with the solvent system H_2_O/MeOH (55:45). Fr. D_19_ (600 mg) was applied to SCC (150 g) using CHCl_3_/MeOH (100:0 to 70:30) and obtained 13 subfractions, D_19a–m_. D_19l_ was applied to the HPLC system at a flow rate of 4 mL/min. The solvent system used was H_2_O/MeOH (50:50). Compound **5** (3.2 mg) was purified.

Fr. E (650 mg) was subjected to SCC (75 g) using CHCl_3_/MeOH (100:0 to 85:15) as the solvent system to give Fr. E_1–22_. Fr. E_7_ (100 mg) was applied to Sephadex LH-20 CC (65 g, MeOH) and obtained 7 subfractions, E_7a–g_. E_7e_ (59 mg) was applied to SCC (30 g) with CHCl_3_/MeOH (100:0 to 92:8) and Compound **6** (16.1 mg) was purified.

### 4.4. In Vivo Assays

#### 4.4.1. Animals

In vivo biological activity studies were performed using male Swiss albino mice and Sprague-Dawley rats. Animals were obtained from Kobay laboratory animal production (Ankara, Türkiye). During this time, the animals were housed in polysulfone cages under controlled conditions at room temperature (20–25 °C) and constant humidity (40–50%), with a 12:12-h light–dark cycle and were provided with standard feed and water. Seven animals were used in each group. Animal experiments were conducted in accordance with the European ethical guidelines for animal experimentation and internationally accepted ethical standards for the care and use of laboratory animals’ approval. This study was carried out in accordance with the approval of the Local Ethics Committee for Animal Experimentation (protocol no. 786).

#### 4.4.2. Preparation of Test Samples

For the evaluation of anti-inflammatory activity, test materials were suspended in a mixture of distilled water and 0.5% sodium carboxymethyl cellulose (CMC) and administered orally to the animals. Animals were divided into 5 groups: a control group, a reference drug group, MeOH extract-treated groups, 80% EtOH extract-treated groups, and aqueous extract-treated groups. Animals in the control group were given only 0.5% CMC, while those in the reference drug group were given indomethacin (10 mg/kg) prepared in 0.5% CMC.

To evaluate the wound healing activity, an ointment base containing 1% glycol stearate/1,2 propylene glycol/liquid paraffin (3:6:1) was used as a carrier. Each test sample was homogeneously mixed with this ointment base and applied topically to the wound area at a dose of 0.5 g. Animals were divided into 6 groups: negative control group, vehicle group, reference drug group, MeOH extract-treated groups, 80% EtOH extract-treated groups, and aqueous extract-treated groups. No treatment was applied to animals in the negative control group; only the ointment base was applied to animals in the vehicle group. Madecassol^®^ (Bayer, Istanbul, Türkiye) ointment containing 1% *Centella asiatica* extract was applied to the animals in the reference drug group.

Wound healing activity was performed using linear incision and circular excision wound models. In the examination of anti-inflammatory activity, the capillary permeability increase model created with acetic acid was used.

#### 4.4.3. Acetic Acid-Induced Capillary Permeability

In order to evaluate the effect of test samples on acetic acid-induced increased vascular permeability in mice, the Whittle (1964) method was applied with some modifications [51]. The test sample was administered orally to the mice at a dose of 0.2 mL per 20 g of body weight. Indomethacin was used as a reference compound at a dose of 10 mg/kg. An amount of 0.1 mL of 4% Evans Blue Dye solution was injected into the tail vein of each mouse 30 min after administration. Following this injection, 10 min later, 0.4 mL of the 0.5% acetic acid solution was administered intraperitoneally. The animals were euthanized by cervical dislocation 20 min after the application. The peritoneum of each animal was opened, the internal organs were washed with distilled water, and peritoneal fluid was collected. Using glass wool, the peritoneal fluid was transferred into 10 mL bottles containing 0.1 M NaOH. The bottle was filled up to 10 mL with distilled water. The absorbance of the dye was measured spectrophotometrically at 590 nm. The degree of extravasation of Evans Blue Dye was measured spectrophotometrically at a wavelength of 590 nm [52,53].

#### 4.4.4. Linear Incision Wound Model

Anesthesia was administered to the rats using 0.15 cc of Ketalar^®^ (Pfizer, New York, NY, USA) and the hair on their backs was shaved. On each side of the dorsal midline, at a distance of 1.5 cm, linear paravertebral wounds measuring 5 cm in length were created using a scalpel. The wounds were closed with three surgical sutures placed 1 cm apart. All animals except the negative control group were treated with topical ointment to the wound area once a day for 9 days. The sutures were removed at the end of the ninth day and the animals were euthanized under anesthesia on the tenth day. To determine the rate of wound healing, the tensile strength of the skin tissue was measured using a tensiometer (Zwick/Roell Z0.5, ZwickRoll GmbH & Co. KG, Ulm, Germany) [54].

#### 4.4.5. Circular Excision Wound Model

Anesthesia was administered to the mice using 0.01 cc of Ketalar^®^ and the hair on their dorsal regions was shaved. Two full-thickness circular excision wounds of 5 mm diameter were created bilaterally on the back of each animal with the help of a biopsy drill. The prepared ointments were topically applied to the wound area every day until complete healing was achieved. The wound healing process was monitored daily with a camera (Fuji, S20 Pro, Fujifilm Co., Tokyo, Japan) and the wound area was measured and evaluated using the images obtained with AutoCAD software (Autodesk, Inc., San Rafael, CA, USA). Wound healing was calculated by taking into account the percentage of reduction compared to the initial wound area. At the end of the tenth day, a tissue samples were taken from each mouse and stored appropriately for histopathological analysis [55].

### 4.5. In Vitro Assays

#### 4.5.1. Cell Culture

The RAW 264.7 cells were kindly provided by Prof. Dr. Hasan Kırmızıbekmez (Department of Pharmacognosy, Faculty of Pharmacy, Yeditepe University, Istanbul, Türkiye). The L929 cells were purchased from the Biota Lab, Istanbul, Türkiye. Both cell lines were cultured in a DMEM medium containing 10% FBS and 1% penicillin-streptomycin.

#### 4.5.2. Cell Viability

For the cytotoxicity evaluation, an adapted version of the MTT assay described by Mossman was used [56]. Cell suspensions were prepared at densities of 5 × 10^5^ cells/mL for the RAW 264.7 cell line and 10^5^ cells/mL for the L929 cell line. Cells were seeded into 96-well plates at 100 µL per well. Plates were incubated at 37 °C for 24 h in an environment containing 5% CO_2_ and 95% humidity. After the incubation period, the culture medium was removed and 100 µL of the test solutions at different concentrations prepared in DMEM were added to each well. The cells were incubated for an additional 48 h. Subsequently, the medium was removed and a fresh medium (100 µL) was added to each well. Next, 10 µL of MTT (5 mg MTT/1 mL PBS) was added to each well and the plates were incubated for 4 h. Following this incubation, the medium was aspirated and 100 µL dimethyl sulfoxide (DMSO) was added to each well to dissolve the formed formazan crystals. The absorbance was measured spectrophotometrically at 577 nm with a reference wavelength of 655 nm [57].

#### 4.5.3. LPS-Induced NO, IL-6, and TNF-α Production in the RAW 264.7 Macrophages

Cells were seeded into 96-well plates at a density of 5 × 10^5^ cells/mL. The plates were incubated at 37 °C in a humidified incubator containing 5% CO_2_ for 24 h. At the end of the incubation period, the medium in each well was aspirated and replaced with 100 μL of the sample solutions at various concentrations. Subsequently, 100 μL of the LPS solution (0.2 μg/mL) was added to each well. The plates were then incubated under the same conditions for another 24 h. After incubation, 100 μL of the medium above the cells from each well was transferred to a new 96-well plate. Nitric oxide (NO) production was determined using the Griess reaction [58], by adding 100 μL of Griess reagent to each well [57]. The levels of IL-6 and TNF-*α* were measured using ELISA kits in accordance with the manufacturer’s instructions, based on the collected medium above the cells [57,59,60]. Indomethacin was used as the reference anti-inflammatory compound in IL-6 and TNF-*α* production experiments.

#### 4.5.4. Scratch Assay

The wound healing effect was determined on the L929 fibroblast cell line using the scratch method. The L929 cells were seeded in 96-well plates at 10^5^ concentrations with 100 μL per well and incubated for 24 h. Afterwards, the cells were scratched with a 200 μL pipette tip, thus creating a wound model in the cells. The medium in the wells was discarded and replaced with 100 μL of fresh medium containing the samples at various concentrations. DMEM without FBS was used as the negative control and DMEM with 10% FBS was used as the positive control. The degree of wound closure in the cells was measured at 0 and 24 h [61].

#### 4.5.5. Antioxidant Activity

The antioxidant activity of the *P. indica* extract and fractions were evaluated by measuring their scavenging effects against the DPPH, ABTS, and SO radicals.

##### Determination of the DPPH Radical Scavenging Activity

DPPH is a stable free radical with an unpaired valence electron at a nitrogen atom bridge. The DPPH radicals are reduced in the presence of antioxidant compounds, allowing for a determination of the radical scavenging activity [62]. This assay is frequently employed for an antioxidant evaluation because it is valid, inexpensive, rapid, and straightforward [63].

Sample solutions were prepared at various concentrations in MeOH. Then, 200 μL of each sample and the MeOH solution (blank) were transferred into a 96-well plate, followed by the addition 50 μL of the 1 mM DPPH solution. After waiting in the dark for 30 min, the absorbance was measured at 520 nm. Quercetin, known for its antioxidant properties, was used as a reference compound. The DPPH radical scavenging activity was assessed by comparing the absorbance of the control solution with that of the sample solutions [27].

##### Determination of the ABTS Radical Scavenging Activity

The ABTS solution (7 mM) prepared in distilled water and in a 2.45 mM potassium persulfate solution were mixed in equal volumes and kept in the dark for 12–16 h. In this way, the ABTS radical (ABTS^+^) was formed and the solution was activated. Dilution was made until the absorbance value of the ABTS^+^ solution at 734 nm was 0.700 ± 0.050. A total of 80% EtOH was used for dilution. Sample solutions were prepared in 24-well plates with 500 μL of 80% EtOH at various concentrations. A total of 130 μL of each solution was transferred to a 96-well plate and 50 μL of ABTS^+^ (absorbance = 0.700 ± 0.050) was added to the wells. Absorbance measurements were performed at 734 nm. Trolox (6-hydroxy-2,5,7,8-tetramethylchroman-2-carboxylic acid) was used as a standard compound in the experiment.

The radical scavenging activity was determined by comparing the absorbance of the solution (blank) containing 80% EtOH and ABTS^+^ radical with the absorbances of the test solutions.

##### Determination of the SO Radical Scavenging Activity

This assay is based on the reduction of the nitro blue tetrazolium (NBT) reagent by superoxide anion radicals generated by DMSO, forming a blue formazan compound. In the presence of antioxidant compounds, the concentration of superoxide radicals decreases, resulting in a change in the amount of the colored diformazan compound. The radical scavenging activity is measured by determining this change using a spectrophotometer.

Sample solutions were prepared at various concentrations in DMSO. A total of 30 μL of the solutions were transferred to 96-well plates followed by the addition of 10 μL of the NBT (nitro blue tetrazolium) solution. Finally, 100 µL of alkaline DMSO, prepared by mixing 0.9 mL of DMSO with 0.1 mL of 5 mM NaOH, was added to each well. An absorbance measurement was made at 560 nm. Quercetin was used as the reference compound.

The SO radical scavenging activity was determined by comparing the absorbance of the control solution (containing DMSO, NBT, and alkaline DMSO) with the absorbance of the sample solutions [27].

### 4.6. Statistical Analysis

Results are presented as the mean of three repeated measurements ± the standard error of the mean (SEM). GraphPad Prism 10.0 program was used for all statistical evaluations. Differences between groups were analyzed using Dunnett’s multiple comparison test after a one-way ANOVA test. A *p* value of less than 0.05 was considered statistically significant.

## 5. Conclusions

This study includes detailed phytochemical and biological activity studies on the aerial parts of *P. indica*. Plantarenaloside (**1**), 3-oxo-*α*-ionol *β*-glucoside (**2**), martynoside (**3**), acteoside (**4**), feruloyl gardoside (**5**), and ursolic acid (**6**) were isolated from an aqueous phase of an 80% EtOH extract of the above-ground parts of *P. indica*. Of these, 3-oxo-*α*-ionol *β*-glucoside (**2**) and feruloyl gardoside (**5**) were determined to have been obtained from a *Plantago* species for the first time. In vitro studies showed that the 80% EtOH extract and the isolated compounds have significant anti-inflammatory and wound-healing potential. Acteoside inhibited NO (65.66%) and IL-6 (64.45%) production to the highest degree, while ursolic acid was the most effective compound in TNF-*α* inhibition (92.43%). In the scratch test, 3-oxo-*α*-ionol *β*-glucoside (12.5 μM; 72.2%) showed the highest wound closure activity. In vivo experiments were conducted on the MeOH, 80% EtOH, and water extracts. These included acetic acid-induced capillary permeability, linear incision wound model, and circular excision wound model experiments. In all three experiments, the 80% EtOH extract showed the strongest effect (35.3%, 26.5%, and 37.7%, respectively). These findings reveal that *P. indica* has considerable potential in terms of anti-inflammatory and wound-healing activities.

## Figures and Tables

**Table 1:** Inhibitory effect of the test materials on inflammation with acetic acid-induced increased capillary permeability.

Material	Extract Type	Dose (mg/kg)	Evans Blue Dye Concentration (μg/mL) ± SEM	Inhibition (%)
Control			10.91 ± 1.13	
*Plantago indica*	MeOH	100	8.01 ± 0.72	26.6 *
	80% EtOH	100	7.06 ± 0.33	35.3 **
	Water	100	9.27 ± 0.29	15.0
Indomethacin		10	5.01 ± 0.39	54.1 ***

*: *p* < 0.05; **: *p* < 0.01; ***: *p* < 0.01; SEM: Standard error of the mean.

**Table 2:** Effect of the test materials on wound healing using a linear incision model.

Material	Extract Type	Tensile Strength ± SEM	(Tensile Strength %)
Vehicle	-	18.25 ± 3.37	-
Negative Control	-	19.73 ± 2.61	-
*Plantago indica*	MeOH	21.82 ± 2.20	19.6
	80% EtOH	23.09 ± 2.05	26.5 *
	Water	17.12 ± 2.07	6.2
Madecassol^®^	-	24.49 ± 1.15	34.2 **

*: *p* < 0.05; **: *p* < 0.01; SEM: Standard error of the mean. The vehicle group was compared to the negative control group; the extracts and the reference material were compared to the vehicle group.

**Table 3:** Effect of the test materials on wound healing using the circular excision model.

Material	Extract Type	Wound Area (mm^2^) ± SEM (Contraction%)						
		Day 0	Day 2	Day 4	Day 6	Day 8	Day 10	Day 12
Vehicle		18.27 ± 2.13	18.34 ± 2.90	16.25 ± 1.86	14.26 ± 1.93	8.21 ± 1.34	5.08 ± 1.23	4.22 ± 0.91
Negative Control		18.34 ± 2.19	19.07 ± 2.46	16.92 ± 2.11	13.67 ± 1.71	9.83 ± 1.62	7.21 ± 1.32	4.80 ± 1.16
*Plantago indica*	MeOH	19.09 ± 3.16	15.02 ± 1.61 (18.1)	14.37 ± 1.58 (11.5)	10.18 ± 1.12 (28.6) *	5.15 ± 1.03 (37.3) *	3.37 ± 0.98 (33.7) *	3.16 ± 0.12 (25.1)
	80% EtOH	18.13 ± 2.24	15.38 ± 2.07 (16.1)	13.51 ± 1.14 (16.8)	9.94 ± 1.36 (30.2) *	5.07 ± 1.16 (38.2) *	3.43 ± 1.05 (32.4) *	2.63 ± 0.38 (37.7) *
	Water	19.07 ± 2.68	17.92 ± 2.08 (2.3)	11.43 ± 1.93 (8.8)	13.49 ± 1.56 (5.3)	7.28 ± 1.53 (11.3)	4.36 ± 0.59 (14.1)	3.71 ± 0.20 (12.0)
Madecassol^®^		18.34 ± 2.01	14.23 ± 2.34 (22.5)	11.37 ± 2.02 (30.0) *	7.16 ± 1.92 (49.7) **	4.03 ± 0.97 (50.9) **	1.18 ± 0.24 (76.8) ***	0.00 ± 0.00 (100.00) ***

*: *p* < 0.05; **: *p* < 0.01; ***: *p* < 0.001; SEM: Standard error of the mean. Percentage of the contraction values: the vehicle group was compared to the negative control group; the extracts and the reference material were compared to the vehicle group.

**Table 4:** Effect of extract and fractions on scratch wound model in the L929 fibroblast (10^5^ cells/mL).

Materials	Statistical Mean ± SEM (Closure %)			
	20 μg/mL	100 μg/mL	200 μg/mL	400 μg/mL
Extract	18.30 ± 1.07	30.83 ± 3.34 **	10.63 ± 0.61	12.32 ± 2.00
	**10 μg/mL**	**50 μg/mL**	**100 μg/mL**	**200 μg/mL**
Fr. A	−0.05 ± 2.13	11.19 ± 0.92	14.31 ± 0.62	7.26 ± 1.69
Fr. B	90.83 ± 5.45 ***	71.70 ± 2.93 ***	58.39 ± 1.25 ***	47.07 ± 6.70 ***
Fr. C	31.25 ± 2.93 **	37.40 ± 2.55 ***	25.55 ± 1.14 **	36.02 ± 1.90 ***
Fr. D	57.89 ± 2.82 ***	11.54 ± 1.52	56.58 ± 2.80 ***	45.47 ± 2.30 ***
Fr. E	3.43 ± 1.77	−0.95 ± 3.09	11.98 ± 1.17	8.99 ± 1.39
FBS (+)	90.04 ± 5.08			
FBS (−)	5.62 ± 1.75			

Data are presented as the mean ± SEM from three independent experiments. FBS (+), positive control; FBS (−), negative control. **: *p* < 0.01; ***: *p* < 0.001.

**Table 5:** Effect of pure compounds on a scratch wound model in the L929 fibroblast (10^5^ cells/mL).

Materials	Statistical Mean ± SEM (Closure %)					
	3.125 μM	6.25 μM	12.5 μM	25 μM	50 μM	100 μM
Act.	35.09 ± 1.20 ***	53.35 ± 1.24 ***	38.82 ± 2.13 ***	50.12 ± 1.12 ***	19.95 ± 0.71 ***	62.58 ± 0.85 ***
Mar.	66.13 ± 1.94 ***	66.11 ± 2.21 ***	57.00 ± 2.14 ***	34.42 ± 1.99 ***	33.89 ± 1.31 ***	14.71 ± 1.11 ***
Pla.	24.99 ± 0.53 ***	44.78 ± 1.55 ***	50.28 ± 0.79 ***	26.62 ± 1.14 ***	29.33 ± 1.10 ***	15.86 ± 0.87 ***
U.A.	17.55 ± 1.02 ***	2.61 ± 0.57	13.36 ± 0.33 *	6.54 ± 0.55	3.07 ± 0.69	13.05 ± 1.08 **
3-oxo.	42.47 ± 1.19 ***	62.71 ± 3.84 ***	75.20 ± 2.00 ***	60.28 ± 2.04 ***	4.69 ± 0.80	30.85 ± 1.60 ***
F.Gar.	26.17 ± 1.17 ***	20.21 ± 0.83 ***	26.16 ± 1.41 ***	21.45 ± 1.32 ***	43.95 ± 1.45 ***	33.80 ± 0.64 ***
FBS (+)	96.84 ± 2.01					
FBS (−)	4.99 ± 1.70					

Data are presented as the mean ± SEM from three independent experiments. FBS (+), positive control; FBS (−), negative control. *: *p* < 0.05; **: *p* < 0.01; ***: *p* < 0.001. Act.: Acteoside; Mar.: Martynoside; Pla.: Plantarenaloside; U.A.: Ursolic Acid; 3-oxo.: 3-oxo-*α*-ionol *β*-glucoside; F.Gar.: Feruloyl Gardoside.

## Data Availability

The data presented in this study are available upon request from the corresponding author. The data are not publicly available due to privacy and ethical restrictions.

## Supplementary Materials

The following supporting information can be downloaded at: https://www.mdpi.com/article/10.3390/plants15010141/s1, Figures S1–S36: NMR (^1^H, ^13^C, COSY, HMQC, HMBC) and chemical structures of compounds 1–6 (plantarenaloside, 3-oxo-*α*-ionol *β*-glucoside, martynoside, acteoside, feruloyl gardoside, ursolic acid).

## Abbreviations

The following abbreviations are used in this manuscript:

ABTS	2,2′-azino-bis(3-ethylbenzothiazoline-6-sulfonic acid)
CMC	Carboxymethyl Cellulose
DMEM	Dulbecco’s Modified Eagle Medium
DMSO	Dimethyl Sulfoxide
DPPH	2,2-Diphenyl-1-picrylhydrazyl
ELISA	Enzyme Linked Immunosorbent Assay
EtOH	Ethanol
FBS	Fetal Bovine Serum
HPLC	High-Performance Liquid Chromatography
IC_50_	Inhibitory Concentration 50%
IL-1*β*	Interleukin-1 beta
IL-6	Interleukin-6
LPS	Lipopolysaccharides
MeOH	Methanol
MTT	3-(4,5-dimethylthiazol-2-yl)-2,5-diphenyl tetrazolium bromide
NaOH	Sodium Hydroxide
NBT	Nitro Blue Tetrazolium
NMR	Nuclear Magnetic Resonance
NO	Nitric oxide
PBS	Phosphate Buffered Saline
SCC	Silica Gel Column Chromatography
SEM	Standard Error of the Mean
SO	Superoxide
TLC	Thin-Layer Chromatography
TNF-*α*	Tumor Necrosis Factor alpha
