# Supplementary material for: Bioactive Metabolites from Aerial Parts of Plantago indica L.: Structural Elucidation and Integrated In Vitro/In Vivo Assessment of Anti-Inflammatory and Wound-Healing Efficacy
Source: Plants (Basel). 2026 Jan 3;15(1):141. doi: 10.3390/plants15010141 (PMC12787502; doi:10.3390/plants15010141)
Supplement: Supplementary file 1 [file plants-15-00141-s001.zip › plants-4035214-supplementary.pdf]

Bioactive Metabolites of Plantago indica L.: Structural Elucidation and Integrated In Vitro/In Vivo Assessment of Anti-inflammatory and Wound-Healing Efficacy

Hilal Bacanak [1,2,*], Zeynep Dogan [1], Esra Küpeli Akkol [3,*], Akito Nagatsu [2] and Iclal Saracoglu [1]

[1] Department of Pharmacognosy, Faculty of Pharmacy, Hacettepe University, Ankara 06100, Türkiye; hilal.bacanak@hacettepe.edu.tr (H.B.); zeynep.ocak@hacettepe.edu.tr (Z.D.); isaracog@hacettepe.edu.tr (I.S.)
[2] Department of Pharmacognosy, College of Pharmacy, Kinjo Gakuin University, Nagoya 463-8521, Japan; anagatsu@kinjo-u.ac.jp
[3] Department of Pharmacognosy, Faculty of Pharmacy, Gazi University, Ankara 06330, Türkiye; esrak@gazi.edu.tr
* Correspondence: hilal.bacanak@hacettepe.edu.tr, ; esrak@gazi.edu.tr

**Table of Contents**	**Page**
**Figure S1:** Chemical Structure of compound (**1**) (plantarenaloside)	3
**Figure S2:** ^{1}H-NMR (600 MHz, CD_3OD) spectrum of compound (**1**) (plantarenaloside)	4
**Figure S3:** ^{13}C-NMR (150 MHz, CD_3OD) spectrum of compound (**1**) (plantarenaloside)	5
**Figure S4:** HMQC spectrum of compound (**1**) (plantarenaloside)	6
**Figure S5:** HMBC spectrum of compound (**1**) (plantarenaloside)	7
**Figure S6:** ^{1}H-^{1}H COSY spectrum of compound (**1**) (plantarenaloside)	8
**Figure S7:** Chemical Structure of compound (**2**) (3-oxo-α-ionol β-glucoside)	9
**Figure S8:** ^{1}H-NMR (600 MHz, CD_3OD) spectrum of compound (**2**) (3-oxo-α-ionol β-glucoside)	10
**Figure S9:** ^{13}C-NMR (150 MHz, CD_3OD) spectrum of compound (**2**) (3-oxo-α-ionol β-glucoside)	11
**Figure S10:** HMQC spectrum of compound (**2**) (3-oxo-α-ionol β-glucoside)	12
**Figure S11:** HMBC spectrum of compound (**2**) (3-oxo-α-ionol β-glucoside)	13
**Figure S12:** ^{1}H-^{1}H COSY spectrum of compound (**2**) (3-oxo-α-ionol β-glucoside)	14
**Figure S13:** Chemical Structure of compound (**3**) (martynoside)	15
**Figure S14:** ^{1}H-NMR (600 MHz, CD_3OD) spectrum of compound (**3**) (martynoside)	16
**Figure S15:** ^{13}C-NMR (150 MHz, CD_3OD) spectrum of compound (**3**) (martynoside)	17
**Figure S16:** HMQC spectrum of compound (**3**) (martynoside)	18
**Figure S17:** HMBC spectrum of compound (**3**) (martynoside)	19
**Figure S18:** ^{1}H-^{1}H COSY spectrum of compound (**3**) (martynoside)	20
**Figure S19:** Chemical Structure of compound (**4**) (acteoside)	21
**Figure S20:** ^{1}H-NMR (600 MHz, CD_3OD) spectrum of compound (**4**) (acteoside)	22
**Figure S21:** ^{13}C-NMR (150 MHz, CD_3OD) spectrum of compound (**4**) (acteoside)	23
**Figure S22:** HMQC spectrum of compound (**4**) (acteoside)	24
**Figure S23:** HMBC spectrum of compound (**4**) (acteoside)	25
**Figure S24:** ^{1}H-^{1}H COSY spectrum of compound (**4**) (acteoside)	26
**Figure S25:** Chemical Structure of compound (**5**) (feruloyl gardoside)	27
**Figure S26:** ^{1}H-NMR (600 MHz, $CDCl_3$) spectrum of compound (**5**) (feruloyl gardoside)	28
**Figure S27:** ^{13}C-NMR (150 MHz, $CDCl_3$) spectrum of compound (**5**) (feruloyl gardoside)	29
**Figure S28:** HMQC spectrum of compound (**5**) (feruloyl gardoside)	30
**Figure S29:** HMBC spectrum of compound (**5**) (feruloyl gardoside)	31
**Figure S30**: HMBC spectrum of compound (**5**) (feruloyl gardoside) (From δ_C 107 ppm to δ_C 152 ppm)	32
**Figure S31:** ^{1}H-^{1}H COSY spectrum of compound (**5**) (feruloyl gardoside)	33
**Figure S32:** Chemical Structure of compound (**6**) (ursolic acid)	34
**Figure S33:** ^{1}H-NMR (600 MHz, CD_3OD) spectrum of compound (**6**) (ursolic acid)	35
**Figure S34:** ^{13}C-NMR (150 MHz, CD_3OD) spectrum of compound (**6**) (ursolic acid)	36
**Figure S35:** HMQC spectrum of compound (**6**) (ursolic acid)	37
**Figure S36:** ^{1}H-^{1}H COSY spectrum of compound (**6**) (ursolic acid)	38

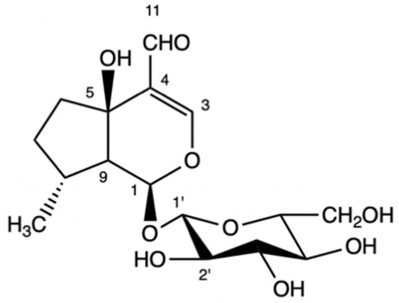

Plantarenaloside (**1**)

Figure S1: Chemical Structure of **1** (plantarenaloside)

Plantarenaloside: $C_{16}H_{24}O_9$ White Amorphous Powder ^{1}H NMR (CD_3OD, 600 MHz); δ_H 5.81 (1H, d, *J* = 1.2 Hz, H-1), 7.37 (1H, s H-3), 1.08 (1H, m, H-6a), 1.84 (1H, m, H-6b), 1.92 (1H, m, H-7a), 2.24 (1H, m, H-7b), 2.39 (1H, m, H-8), 2.44 (1H, m, H-9), 0.90 (3H, d, *J* = 7.2 Hz, H-10), 9.21 (1H, s, H-11), 4.57 (1H, d, *J* = 7.8 Hz, H-1'), 3.14 (1H, dd, *J* = 7.8/9.0 Hz, H-2'), 3.34 (1H, t, *J* = 9.0 Hz, H-3'), 3.21 (1H, t, *J* = 9.6 Hz, H-4'), 3.26 (1H, m, H-5'), 3.61 (1H, dd, *J* = 6.6/12.0 Hz, H-6a'), 3.87 (1H, dd, *J* = 1.8/12.0 Hz, H-6b'), ^{13}C NMR (150 MHz, CD_3OD); δ_C 97.1 (C-1), 164.5 (C-3), 126.7 (C-4), 73.8 (C-5), 39.2 (C-6), 33.3 (C-7), 35.5 (C-8), 52.9 (C-9), 16.7 (C-10), 192.9 (C-11), 100.2 (C-1'), 74.7 (C-2'), 78.8 (C-3'), 72.0 (C-4'), 77.8 (C-5'), 63.1 (C-6').; ESI-MS *m/z* 383.3 $[M + Na]^+$. Data were compared with the literature [1].

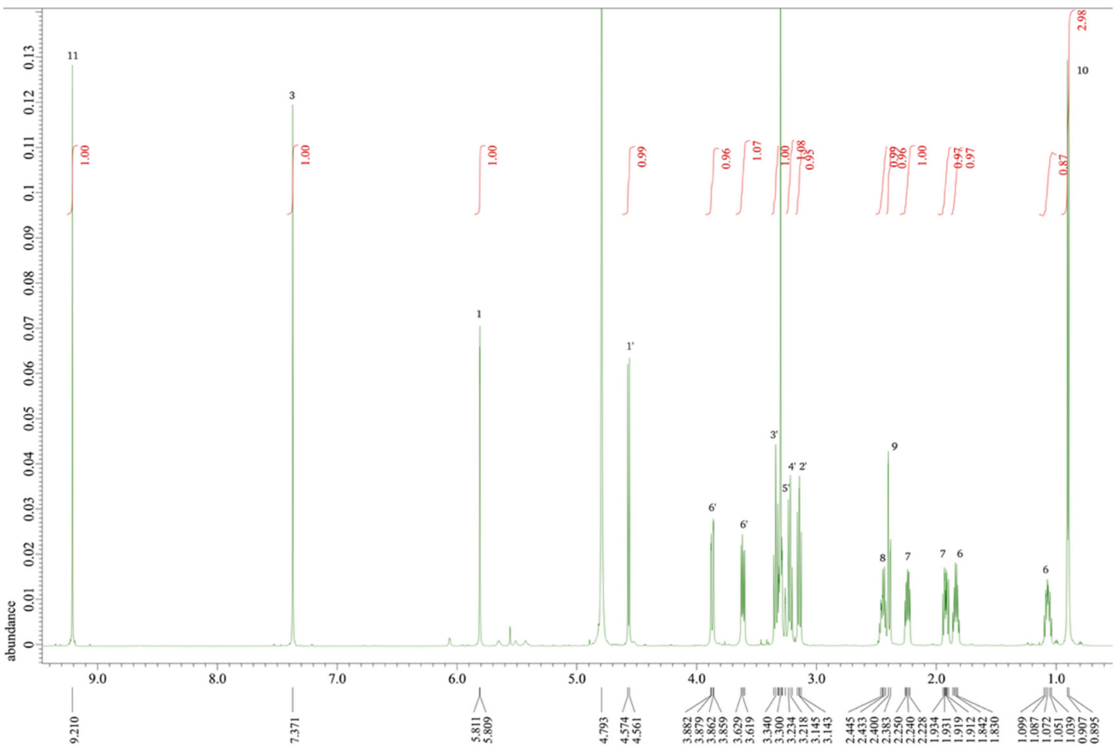

Figure S2: ^{1}H-NMR (600 MHz, CD_3OD) spectrum of **1** (plantarenaloside)

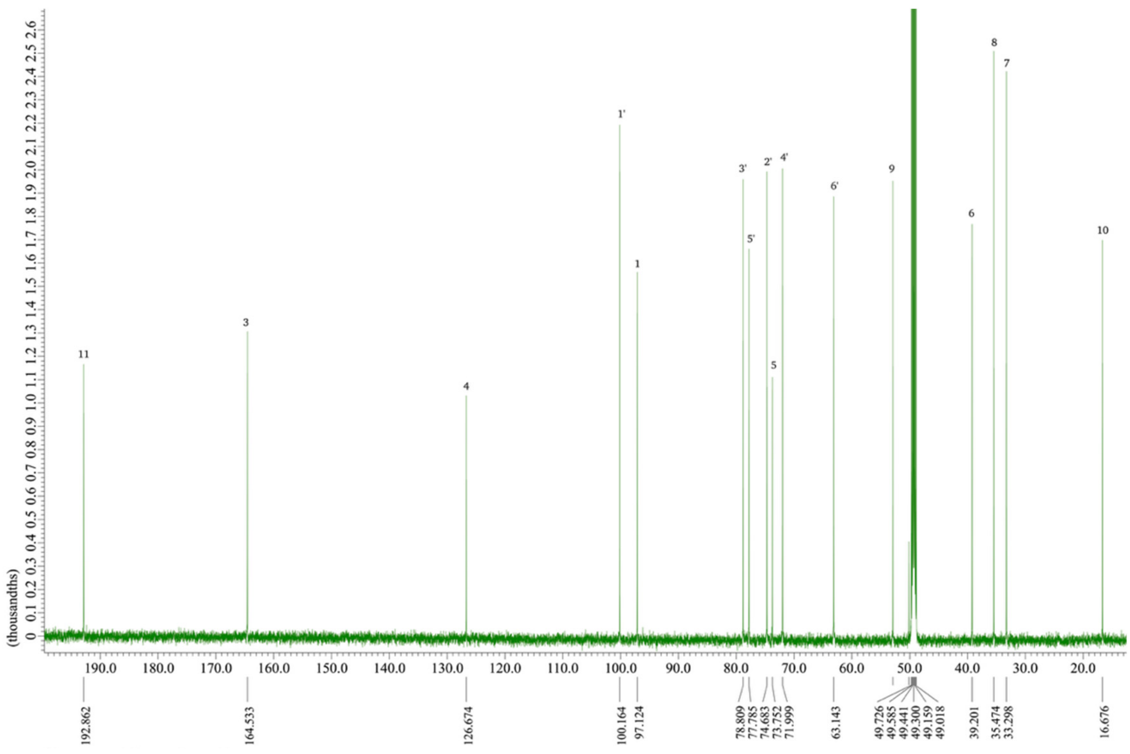

Figure S3: ^{13}C-NMR (150 MHz, CD_3OD) spectrum of **1** (plantarenaloside)

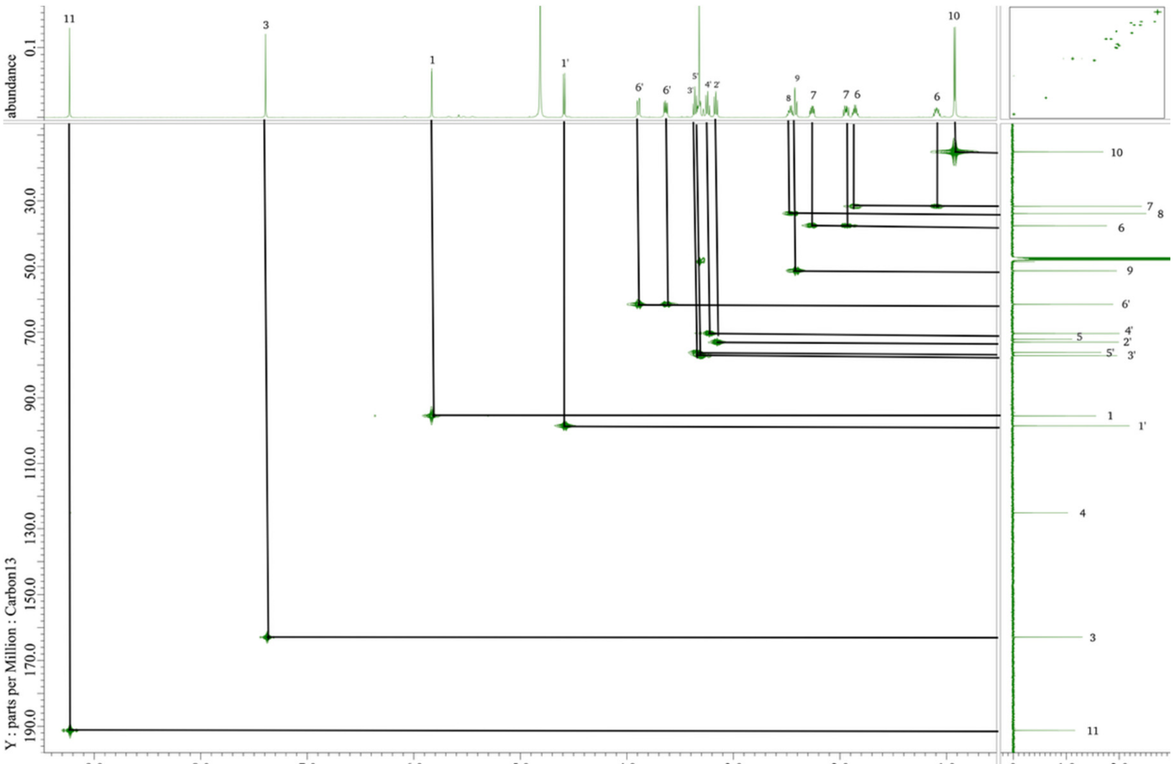

Figure S4: HMQC spectrum of **1** (plantarenaloside)

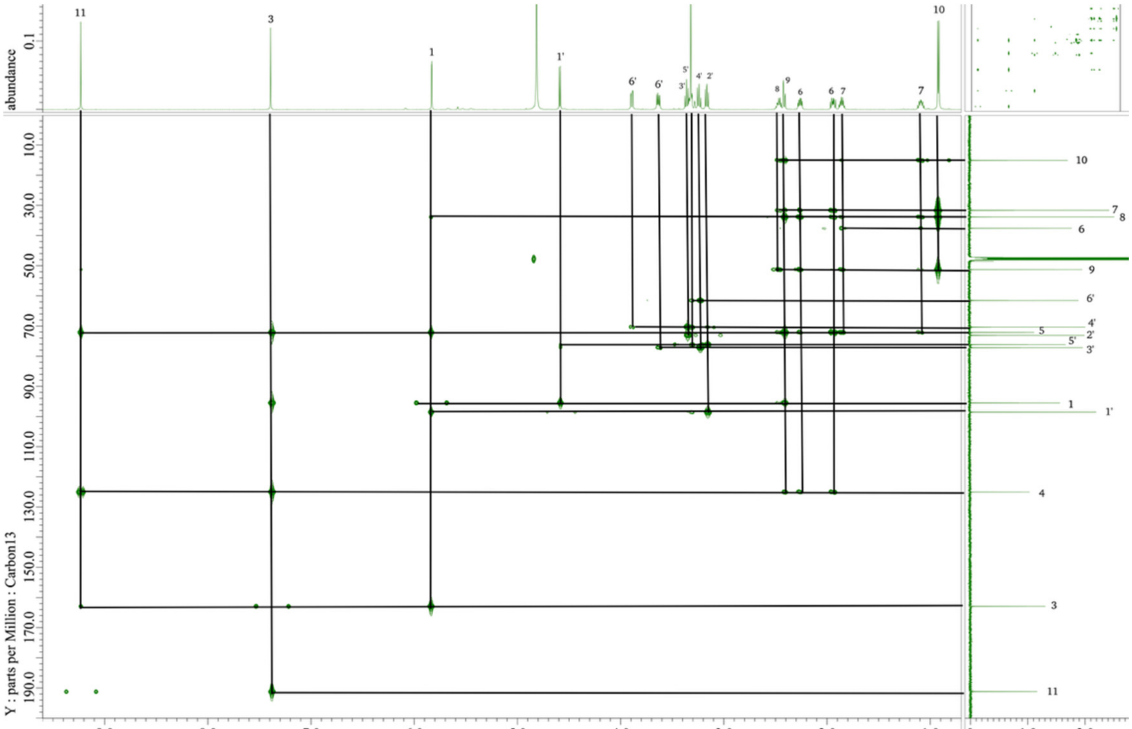

Figure S5: HMBC spectrum of **1** (plantarenaloside)

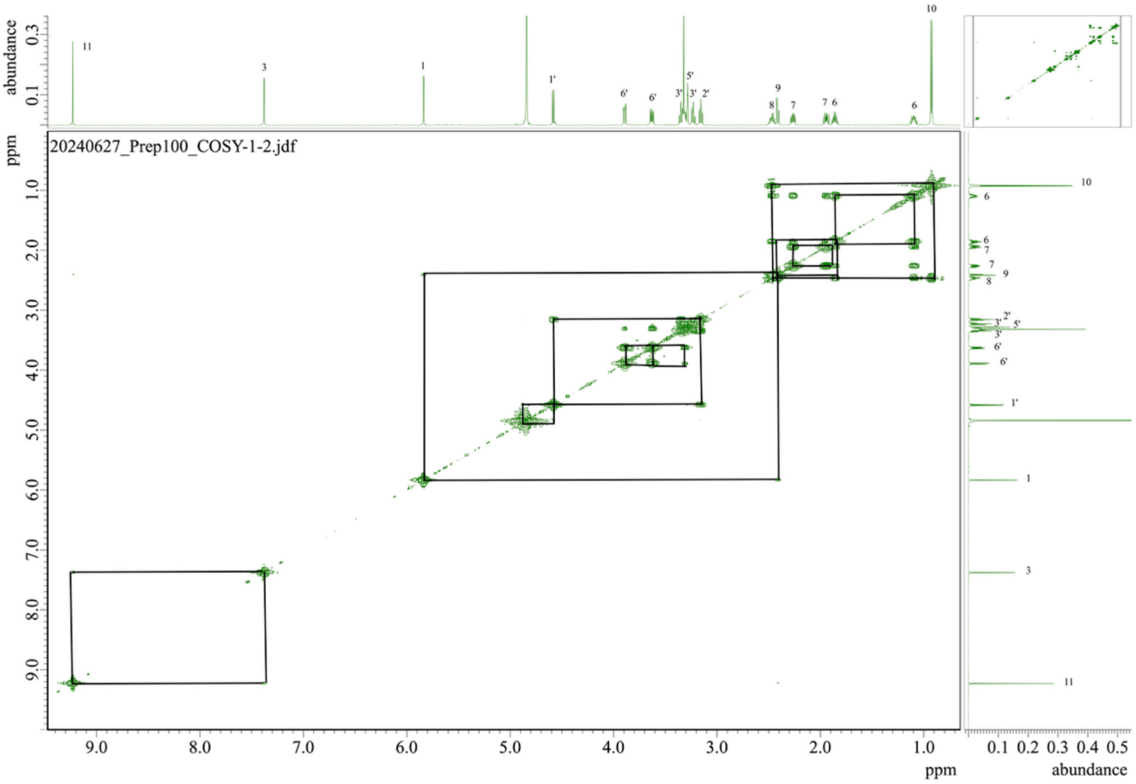

Figure S6: ^{1}H-^{1}H COSY spectrum of **1** (plantarenaloside)

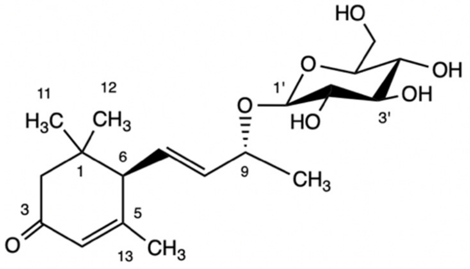

3-oxo-*α*-ionol *β*-glucoside (**2**)

Figure S7: Chemical Structure of **2** (3-oxo-*α*-ionol *β*-glucoside)

3-oxo-*α*-ionol *β*-glucoside: $C_{19}H_{30}O_7$ White Amorphous Powder ^{1}H NMR (CD_3OD, 600 MHz) δ_H 2.04 (1H, d, *J* = 16.8 Hz, H-2a), 2.43 (1H, d, *J* = 16.8 Hz, H-2b), 5.87 (1H, s, H-4), 2.67 (1H, d, *J* = 9.0, H-6), 5.64 (1H, dd, *J* = 9.0/15.0 Hz, H-7), 5.77 (1H, dd, *J* = 6.6/15.6 Hz, H-8), 4.39 (1H, t, *J* = 6.6 Hz, H-9), 1.29 (3H, d, *J* = 4.8 Hz, H-10), 1.02 (3H, s, H-11), 1.00 (3H, s, H-12), 1.93 (3H, brs, H-13), 4.34 (1H, d, *J* = 7.8 Hz, H-1'), 3.17 (1H, t, *J* = 7.8 Hz, H-2'), 3.34 (1H, d, *J* = 5.4 Hz, H-3'), 3.29 (1H, s, H-4'), 3.22 (1H, m, H-5'), 3.65 (1H, dd, *J* = 4.8/12.0 Hz, H-6a'), 3.81 (1H, dd, *J* = 2.4/12.0 Hz, H-6b'), ^{13}C NMR (150 MHz, CD_3OD); δ_C 37.6 (C-1), 48.6 (C-2), 202. 5 (C-3), 126.6 (C-4), 166.4 (C-5), 57.2 (C-6), 129.3 (C-7), 138.7 (C-8), 77.4 (C-9), 21.5 (C-10), 28.0 (C-11), 28.5 (C-12), 24.2 (C-13), 102.9 (C-1'), 75.7 (C-2'), 78.5 (C-3'), 71.9 (C-4'), 78.4 (C-5'), 63.1 (C-6').; ESI-MS *m/z* 415.2 [M + HCOO]$^-$. Data were compared with the literature [2].

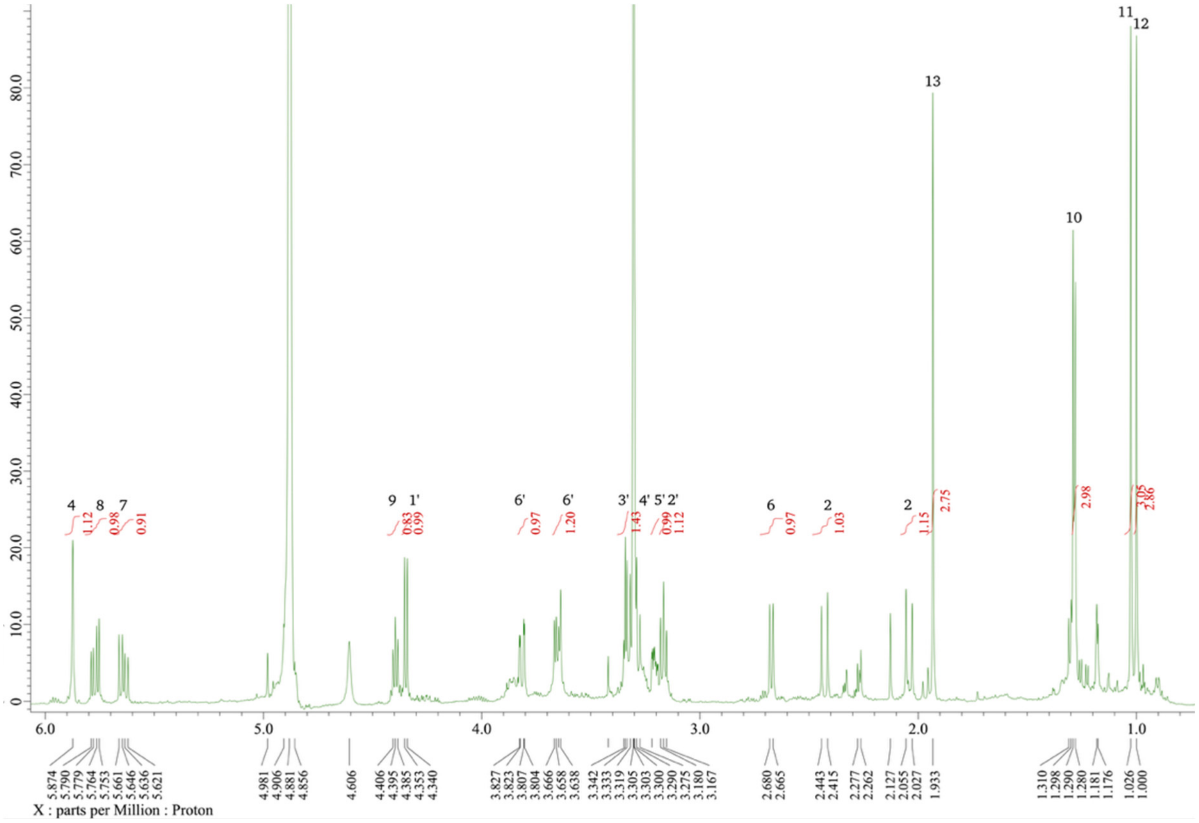

Figure S8: ^{1}H-NMR (600 MHz, CD_3OD) spectrum of **2** (3-oxo-α-ionol β-glucoside)

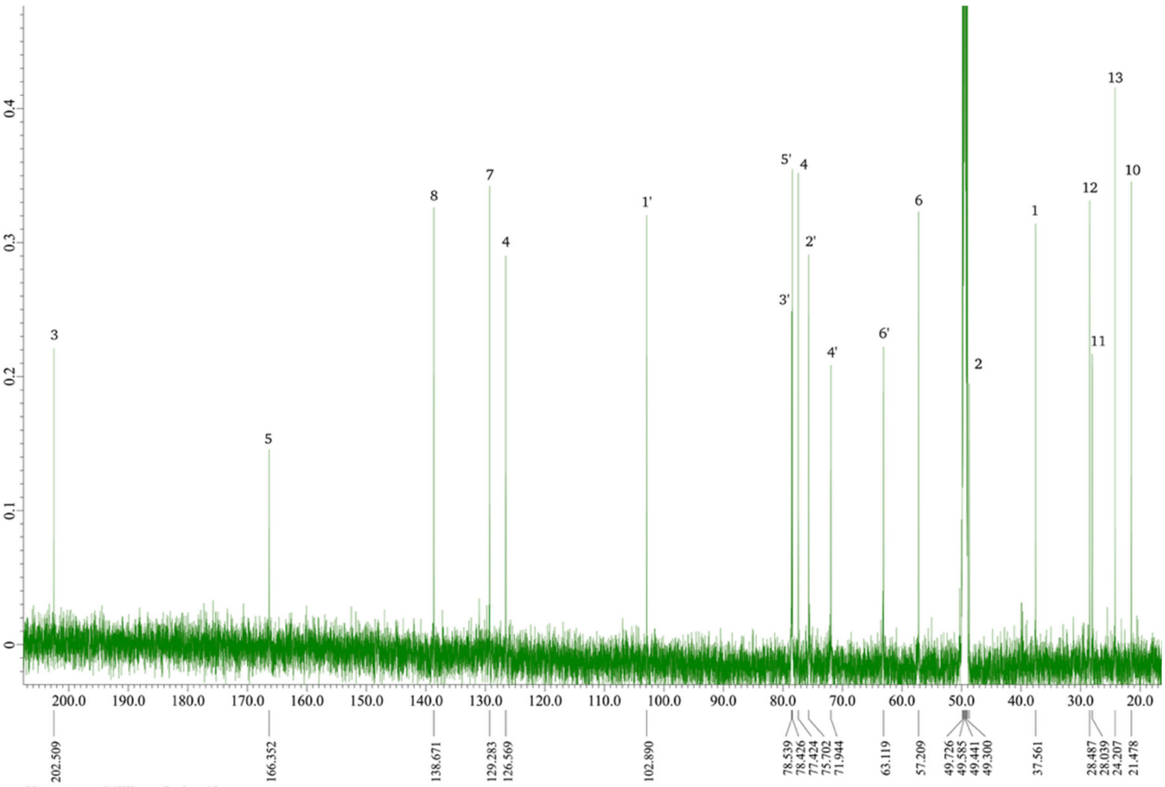

Figure S9: ^{13}C-NMR (150 MHz, CD_3OD) spectrum of **2** (3-oxo-α-ionol β-glucoside)

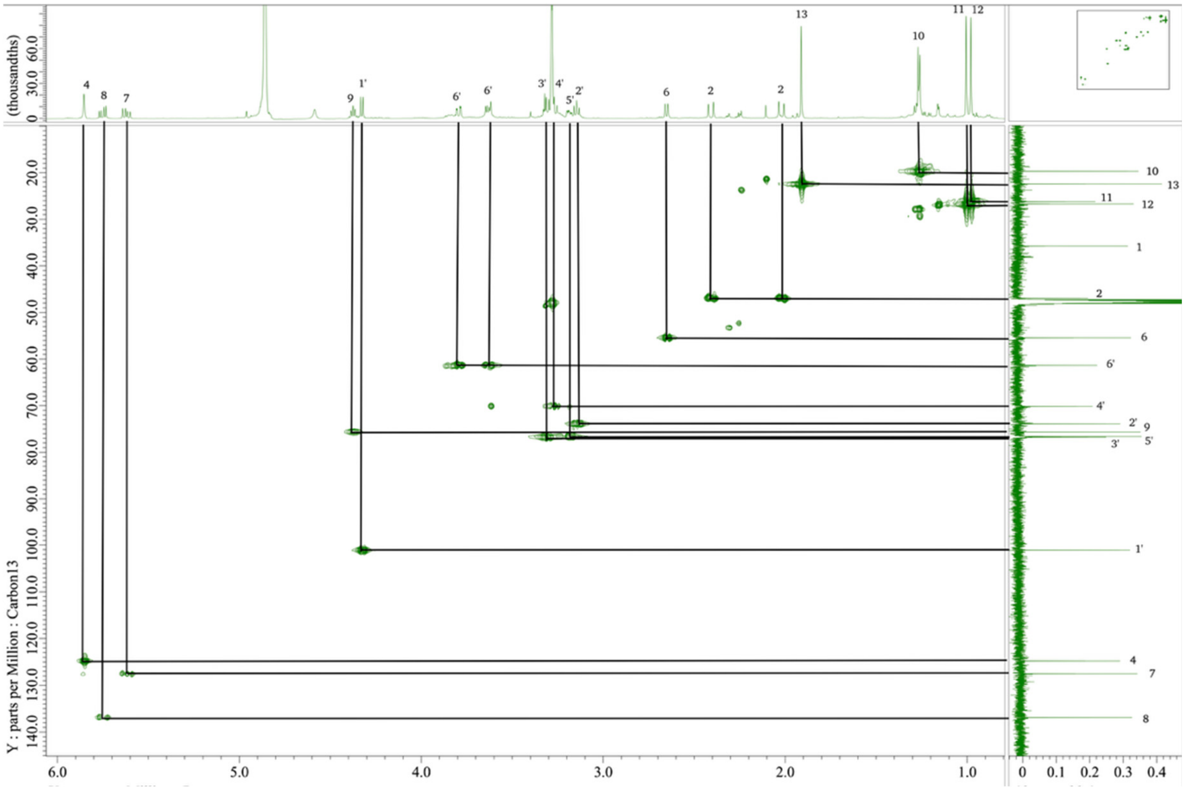

Figure S10: HMQC spectrum of **2** (3-oxo-α-ionol β-glucoside)

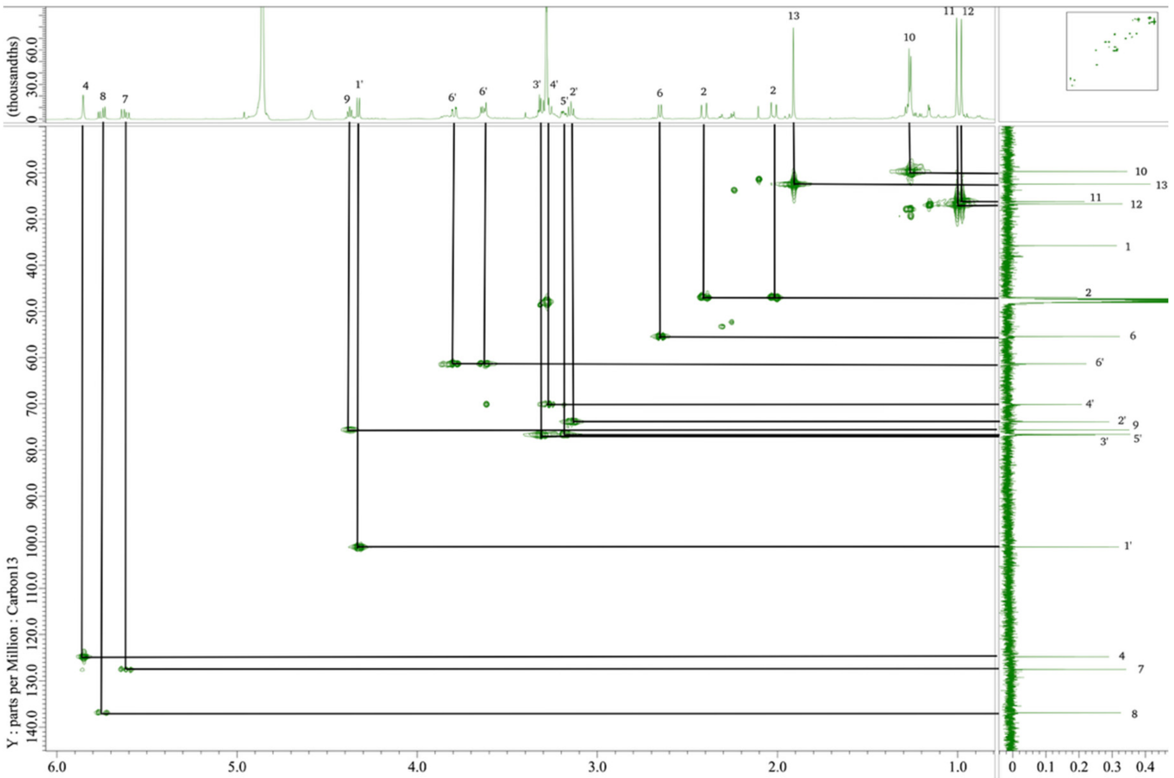

Figure S11: HMBC spectrum of **2** (3-oxo-α-ionol β-glucoside)

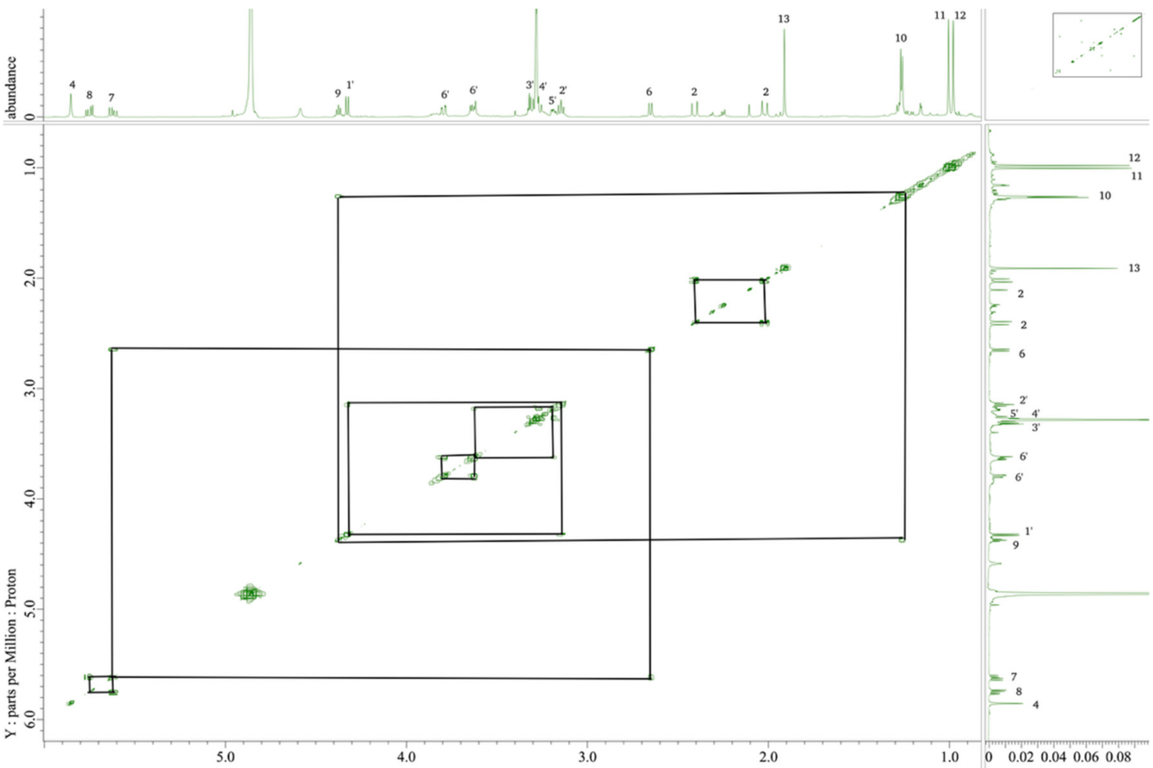

Figure S12: ^{1}H-^{1}H COSY spectrum of **2** (3-oxo-α-ionol β-glucoside)

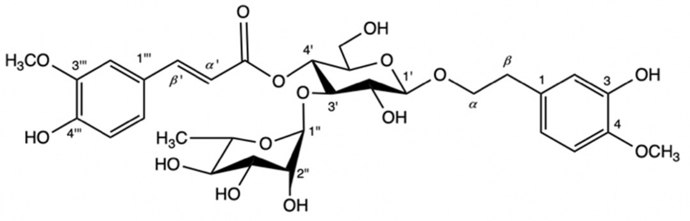

Martynoside (**3**)

Figure S13: Chemical Structure of **3** (martynoside)

Martynoside: $C_{31}H_{40}O_{15}$ White Amorphous Powder ^{1}H NMR (CD_3OD, 600 MHz); δ_H 6.73 (1H, brs, H-2), 6.80 (1H, d, J = 8.0 Hz, H-5), 6.68 (1H, brd, J = 8.4 Hz, H-6), 3.73 (1H, m, H-α1), 4.06 (1H, m, H-α2), 2.82 (1H, m, H-β), 3.81 (3H, s, -OCH_3), 4.37 (1H, d, J = 7.8 Hz, H-1'), 3.39 (1H, brt, J = 8.4 Hz, H-2'), 3.83 (1H, brs, H-3'), 4.92 (1H, brd, J = 9.6 Hz, H-4'), 3.52† (1H, H-5'), 3.51 (1H, dd, J = 6.6/11.0 Hz, H-6'a), 3.62 (1H, brd, J = 4.8 Hz, H-6'b), 5.19 (1H, brs, H-1''), 3.91 (1H, brs, H-2''), 3.56 (1H, brd, J = 3.6 Hz, H-3''), 3.28 (1H, d, J = 9.6 Hz, H-4''), 3.58 (1H, brt, J = 6.0 Hz, H-5''), 1.09 (3H, d, J = 6.2 Hz, H-6''), 7.20 (1H, brs, H-2'''), 6.82 (1H, d, J = 8.4 Hz, H-5'''), 7.08 (1H, brd, J = 7.8 Hz, H-6'''), 6.37 (1H, d, J = 16.0 Hz, H-α'), 7.65 (1H, d, J = 16.0 Hz, H-β'), 3.88 (3H, s, -OCH_3), ^{13}C NMR (150 MHz, CD_3OD); δ_c 133.2 (C-1), 117.4 (C-2), 147.7 (C-3), 147.9 (C-4), 113.2 (C-5), 121.5 (C-6), 72.4 (C-α), 36.9 (C-β), 56.8 (-OCH_3), 104.5 (C-1'), 76.5 (C-2'), 81.8 (C-3'), 70.9 (C-4'), 76.4 (C-5'), 62.7 (C-6'), 103.3 (C-1''), 72.7 (C-2''), 72.4 (C-3''), 74.1 (C-4''), 70.7 (C-5''), 18.7 (C-6''). 128.0 (C-1'''), 112.1 (C-2'''), 149.7 (C-3'''), 151.1 (C-4'''),116.8 (C-5'''), 124.7 (C-6''') 115.4 (C-α'), 148.2 (C-β'), 168,5 (C=O), 56.7 (-OCH_3).; ESI-MS *m/z* 651.2 $[M - H]^-$. Data were compared with the literature [3].

†: Signal patterns are unclear due to overlapping.

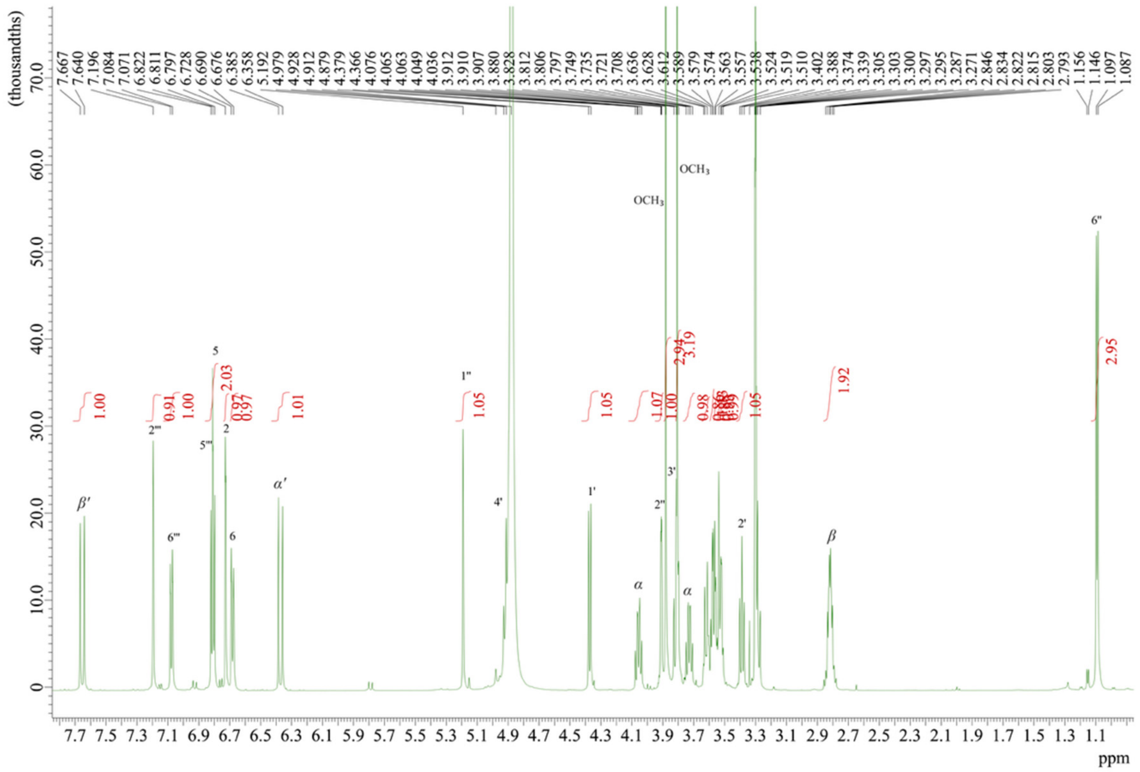

Figure S14: ^{1}H-NMR (600 MHz, CD_3OD) spectrum of **3** (martynoside)

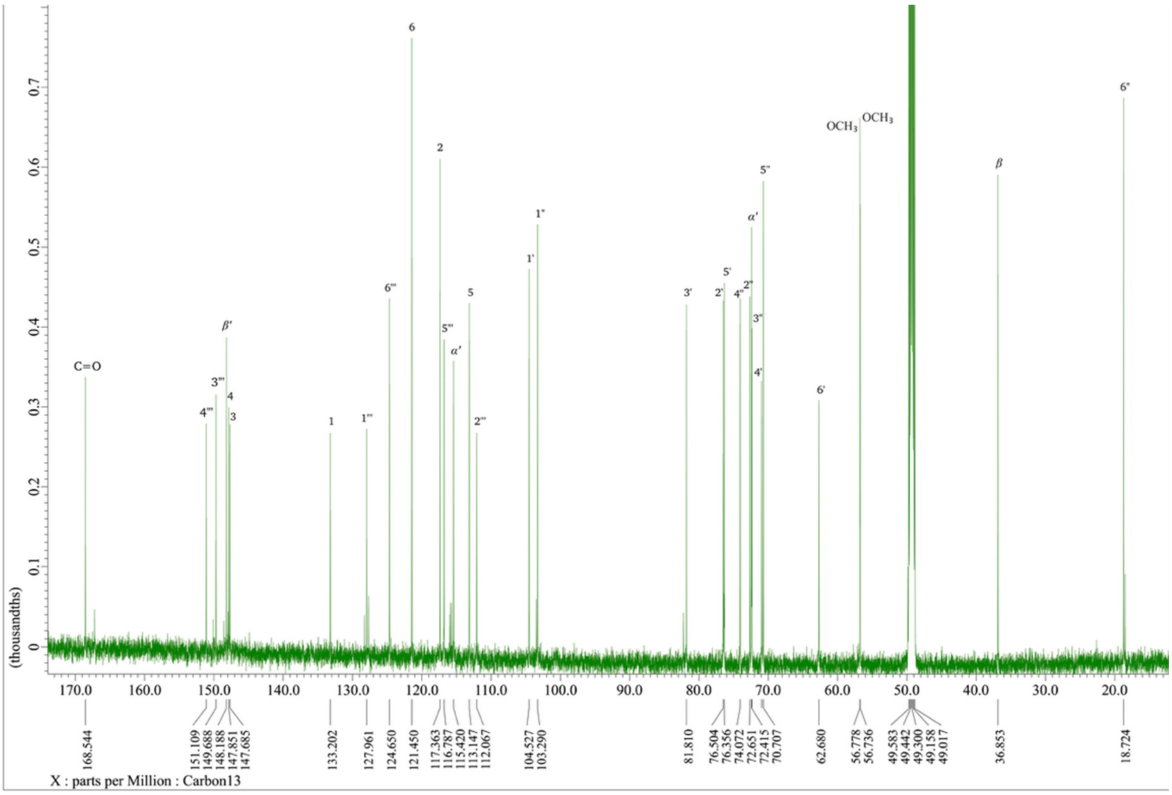

Figure S15: ^{13}C-NMR (150 MHz, CD_3OD) spectrum of **3** (martynoside)

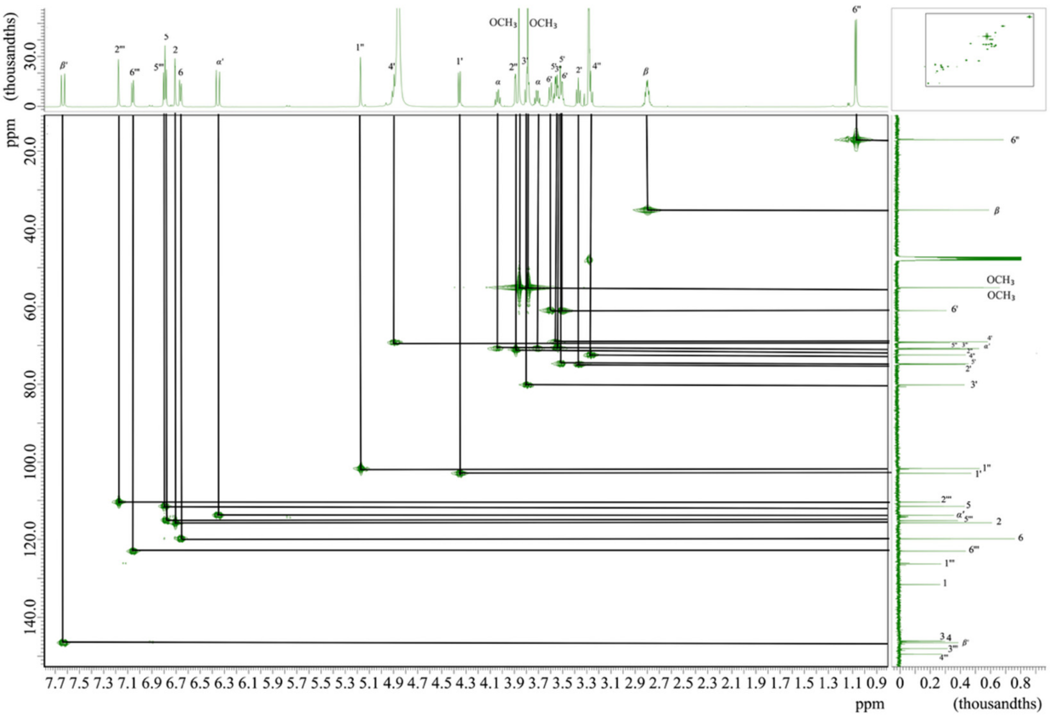

Figure S16: HMQC spectrum of **3** (martynoside)

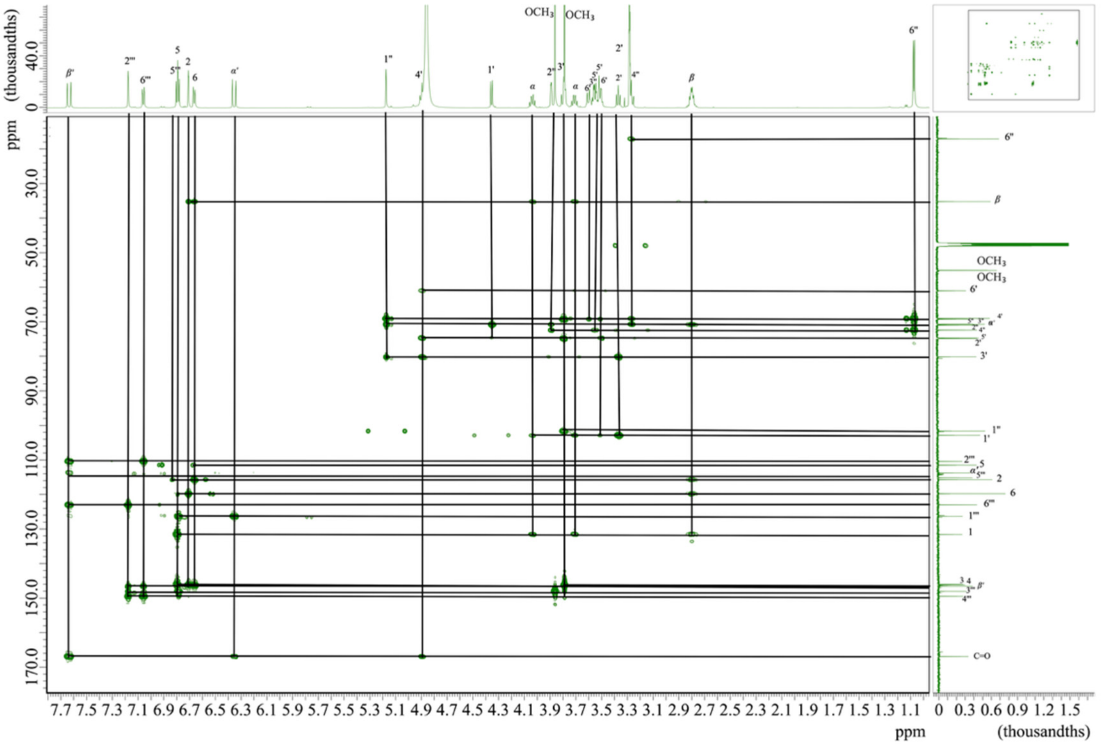

Figure S17: HMBC spectrum of **3** (martynoside)

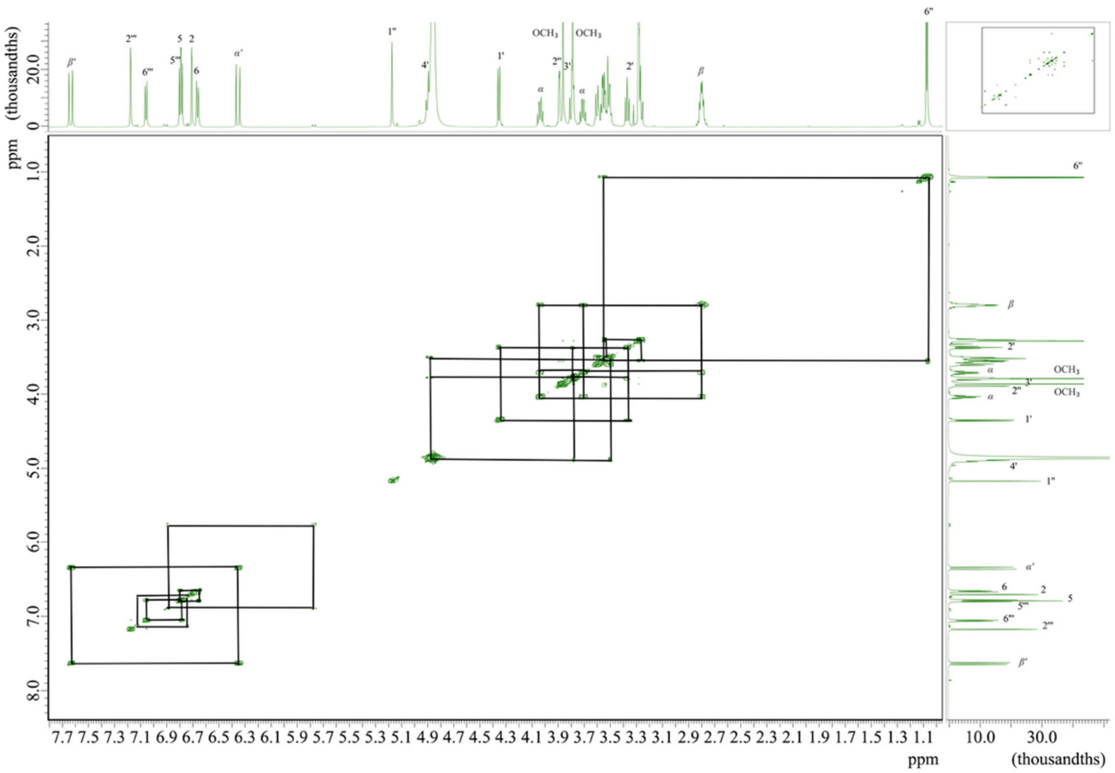

Figure S18: ^{1}H-^{1}H COSY spectrum of **3** (martynoside)

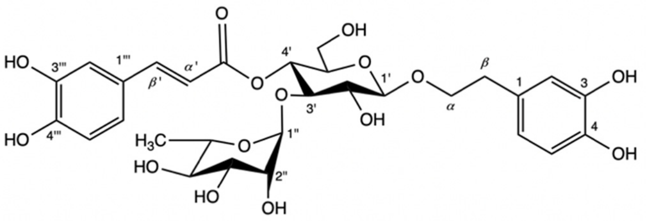

Acteoside (**4**)

Figure S19: Chemical Structure of **4** (acteoside)

Acteoside: $C_{29}H_{36}O_{15}$ White Amorphous Powder ^1H NMR (CD_3OD, 600 MHz); δ_H 6.68 (1H, d, *J* = 1.8 Hz, H-2), 6.66 (1H, d, *J* = 7.8 Hz, H-5), 6.56 (1H, dd, *J* = 1.8/7.8 Hz H-6), 3.71 (1H, t, *J* = 9.0 Hz, H-α1), 4.04 (1H, m, H-α2), 2.79 (1H, m, H-β), 4.37 (1H, d, *J* = 7.8 Hz, H-1'), 3.38 (1H, brt, *J* = 9.0 Hz, H-2'), 3.80 (1H, t, *J* = 9.6 Hz, H-3'), 4.91 (1H, t, *J* = 9.6 Hz, H-4'), 3.53 (1H, m, H-5'), 3.50 (1H, brd, *J* = 5.4 Hz, H-6α'), 3.61 (1H,brd, *J* = 4.8/15.0 Hz, H-6α'), 5.18 (1H, d, *J* = 1.2 Hz, H-1''), 3.90 (1H, brt, *J* = 3.0 Hz, H-2''), 3.56 (1H, dd, *J* = 3.6/9.6 Hz, H-3''), 3.27 (1H, brd, *J* = 9.0 Hz, H-4''), 3.55 (1H, m, H-5''), 1.08 (3H, d, *J* = 6.6 Hz, H-6''), 7.04 (1H, d, *J* = 1.8 Hz, H-2'''), 6.76 (1H, d, *J* = 8.4 Hz, H-5'''), 6.95 (1H, dd, *J* = 2.4/8.4 Hz, H-6'''), 6.26 (1H, d, *J* = 16.0 Hz, H-α'), 7.58 (1H, d, *J* = 16.0 Hz, H-β') ^{13}C NMR (150 MHz, CD_3OD); δc 131.8 (C-1), 117.4 (C-2), 146.5 (C-3), 145.0 (C-4), 116.8 (C-5), 121.5 (C-6), 72.6 (C-α), 36.9 (C-β), 104.5 (C-1'), 76.5 (C-2'), 82.0 (C-3'), 70.7 (C-4'), 76.4 (C-5'), 62.7 (C-6'). 103.3 (C-1''), 72.7 (C-2''), 72.4 (C-3''), 74.1 (C-4''), 70.8 (C-5''), 18.8 (C-6''), 127.9 (C-1'''), 115.5 (C-2'''), 147.2 (C-3'''), 150.2 (C-4'''), 116.6 (C-5'''), 123.5 (C-6''') 115.0 (C-α'), 148.3 (C-β'), 168,6 (C=O).; ESI-MS *m/z* 623.2 $[M - H]^-$. Data were compared with the literature [4]

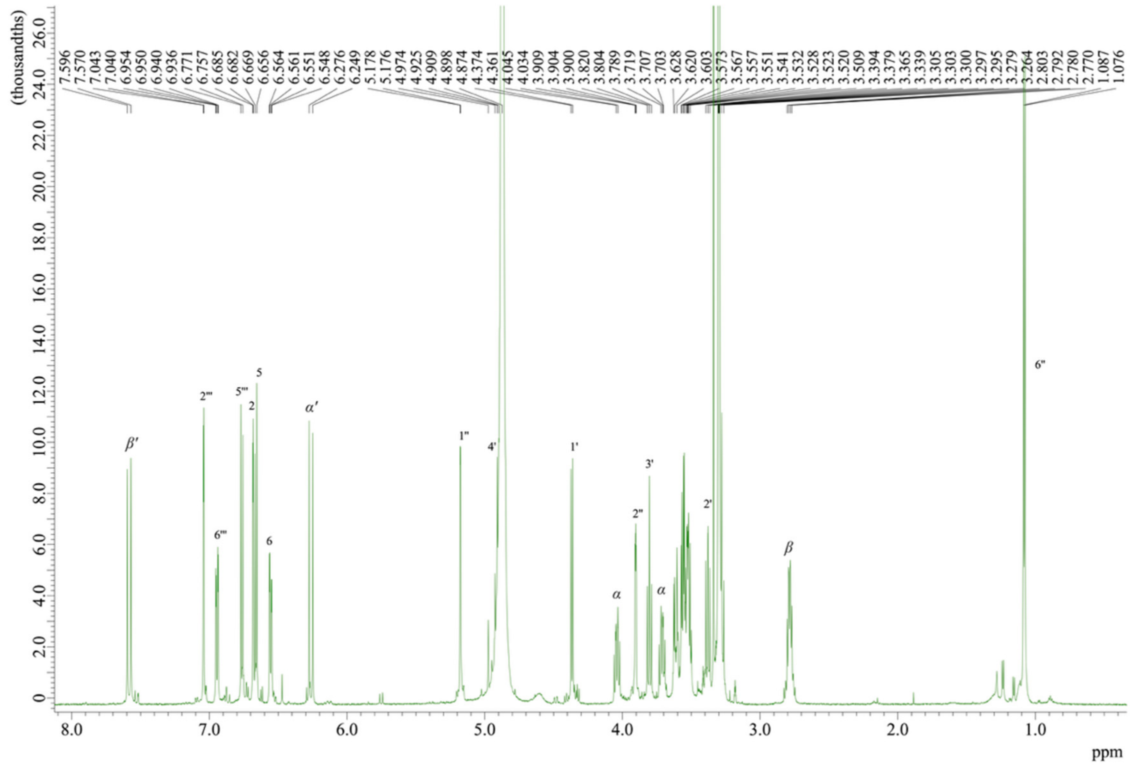

Figure S20: ^{1}H-NMR (600 MHz, CD_3OD) spectrum of **4** (acteoside)

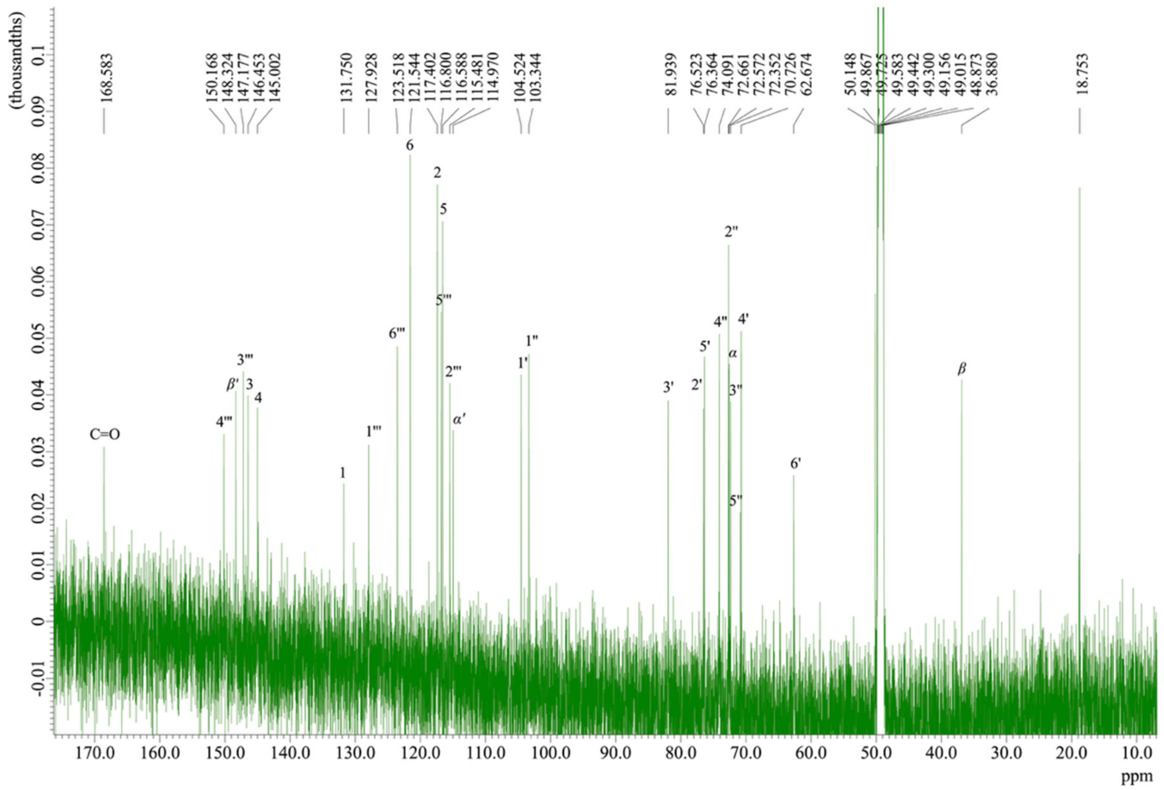

Figure S21: ^{13}C-NMR (150 MHz, CD_3OD) spectrum of **4** (acteoside)

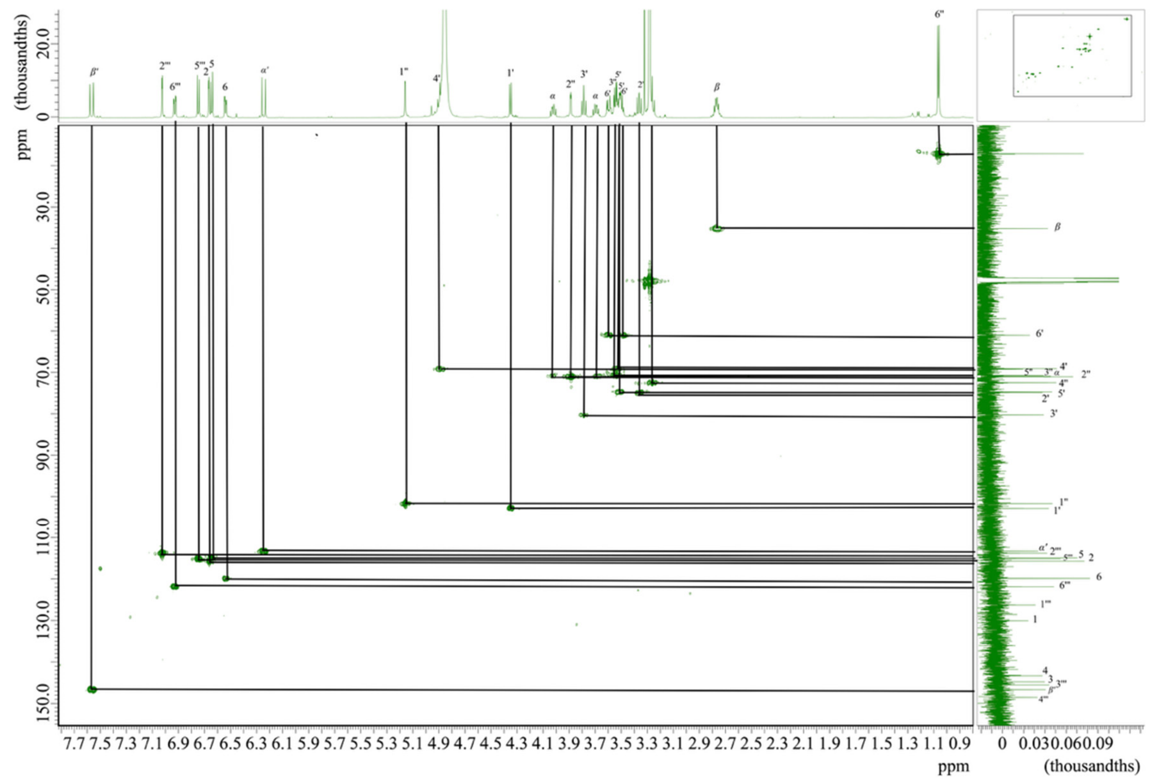

Figure S22: HMQC spectrum of **4** (acteoside)

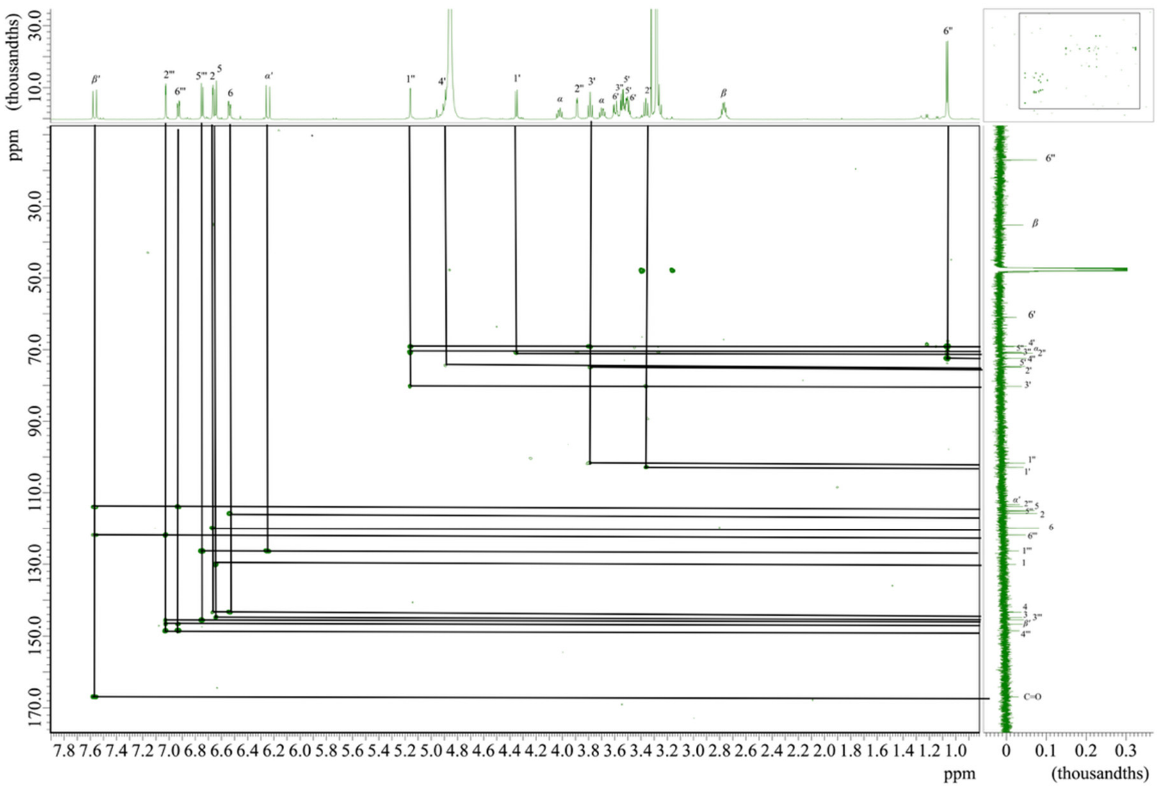

Figure S23: HMBC spectrum of **4** (acteoside)

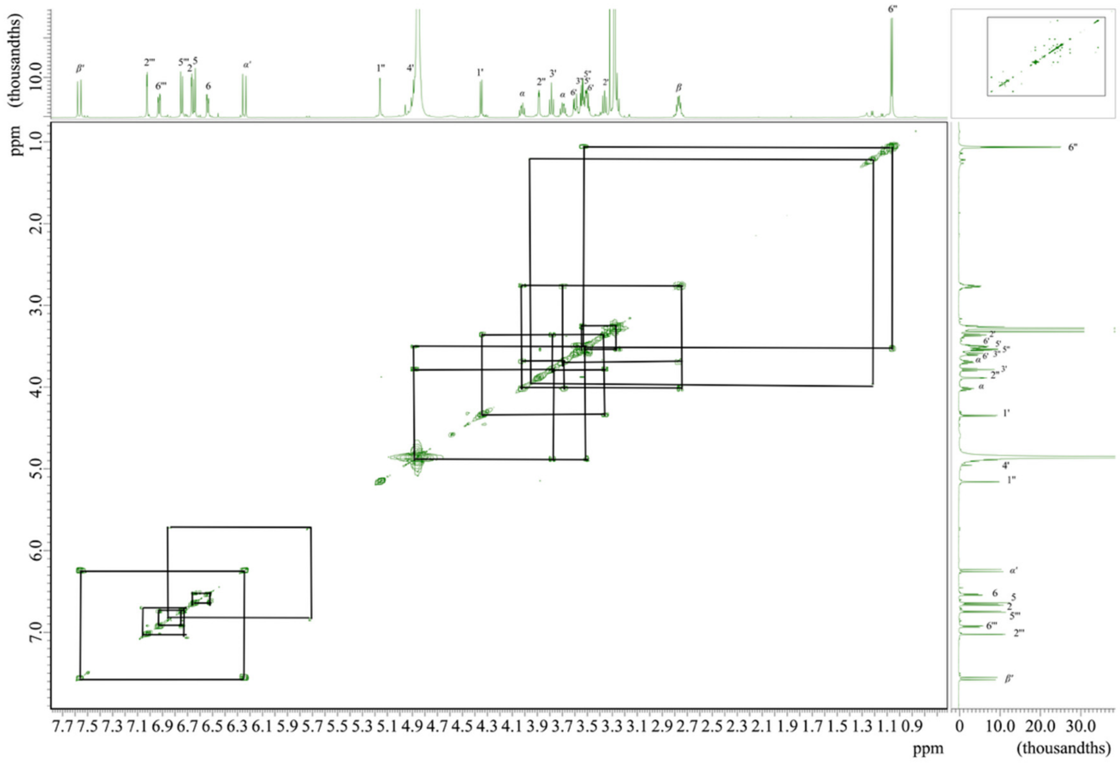

Figure S24: ^1H-^1H COSY spectrum of **4** (acteoside)

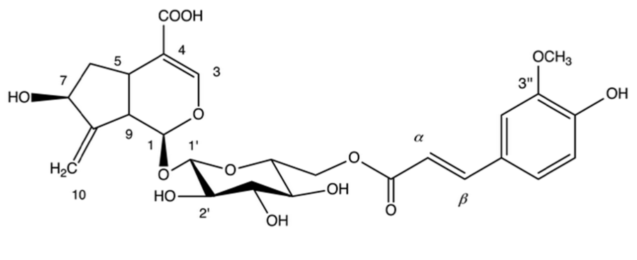

Figure S25: Chemical Structure of **5** (feruloyl gardoside)

Feruloyl Gardoside: $C_{29}H_{36}O_{15}$ White Amorphous Powder ^{1}H NMR (CD_3OD, 600 MHz); δ_H 6.68 (1H, d, *J* = 1.8 Hz, H-2), 6.66 (1H, d, *J* = 7.8 Hz, H-5), 6.56 (1H, dd, *J* = 1.8/7.8 Hz H-6), 3.71 (1H, t, *J* = 9.0 Hz, H-*α*1), 4.04 (1H, m, H-*α*2), 2.79 (1H, m, H-*β*), 4.37 (1H, d, *J* = 7.8 Hz, H-1'), 3.38 (1H, brt, *J* = 9.0 Hz, H-2'), 3.80 (1H, t, *J* = 9.6 Hz, H-3'), 4.91 (1H, t, *J* = 9.6 Hz, H-4'), 3.53 (1H, m, H-5'), 3.50 (1H, brd, *J* = 5.4 Hz, H-6*α*'), 3.61 (1H,brd, *J* = 4.8/15.0 Hz, H-6*α*'), 5.18 (1H, d, *J* = 1.2 Hz, H-1''), 3.90 (1H, brt, *J* = 3.0 Hz, H-2''), 3.56 (1H, dd, *J* = 3.6/9.6 Hz, H-3''), 3.27 (1H, brd, *J* = 9.0 Hz, H-4''), 3.55 (1H, m, H-5''), 1.08 (3H, d, *J* = 6.6 Hz, H-6''), 7.04 (1H, d, *J* = 1.8 Hz, H-2'''), 6.76 (1H, d, *J* = 8.4 Hz, H-5'''), 6.95 (1H, dd, *J* = 2.4/8.4 Hz, H-6'''), 6.26 (1H, d, *J* = 16.0 Hz, H-*α*'), 7.58 (1H, d, *J* = 16.0 Hz, H-*β*') ^{13}C NMR (150 MHz, CD_3OD); δc 131.8 (C-1), 117.4 (C-2), 146.5 (C-3), 145.0 (C-4), 116.8 (C-5), 121.5 (C-6), 72.6 (C-*α*), 36.9 (C-*β*), 104.5 (C-1'), 76.5 (C-2'), 82.0 (C-3'), 70.7 (C-4'), 76.4 (C-5'), 62.7 (C-6'). 103.3 (C-1''), 72.7 (C-2''), 72.4 (C-3''), 74.1 (C-4''), 70.8 (C-5''), 18.8 (C-6''), 127.9 (C-1'''), 115.5 (C-2'''), 147.2 (C-3'''), 150.2 (C-4'''), 116.6 (C-5'''), 123.5 (C-6''') 115.0 (C-*α*'), 148.3 (C-*β*'), 168,6 (C=O).; ESI-MS *m/z* 623.2 $[M - H]^-$. Data were compared with the literature [5].

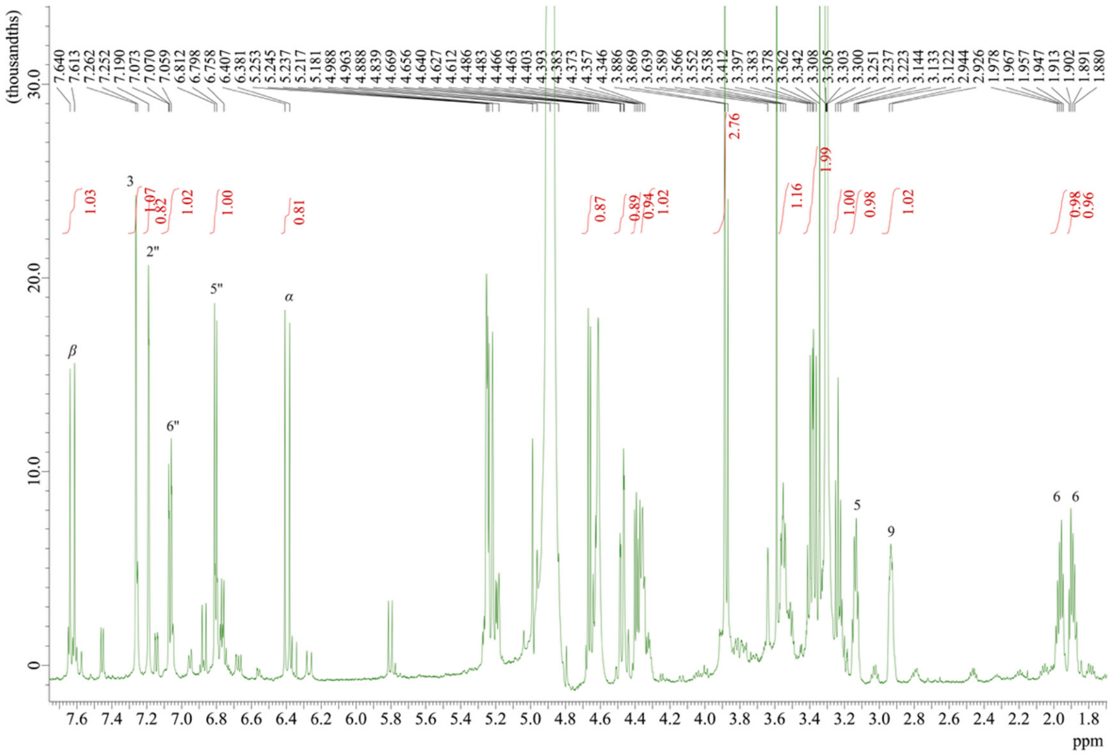

Figure S26: ^{1}H-NMR (600 MHz, CD_3OD) spectrum of **5** (feruloyl gardoside)

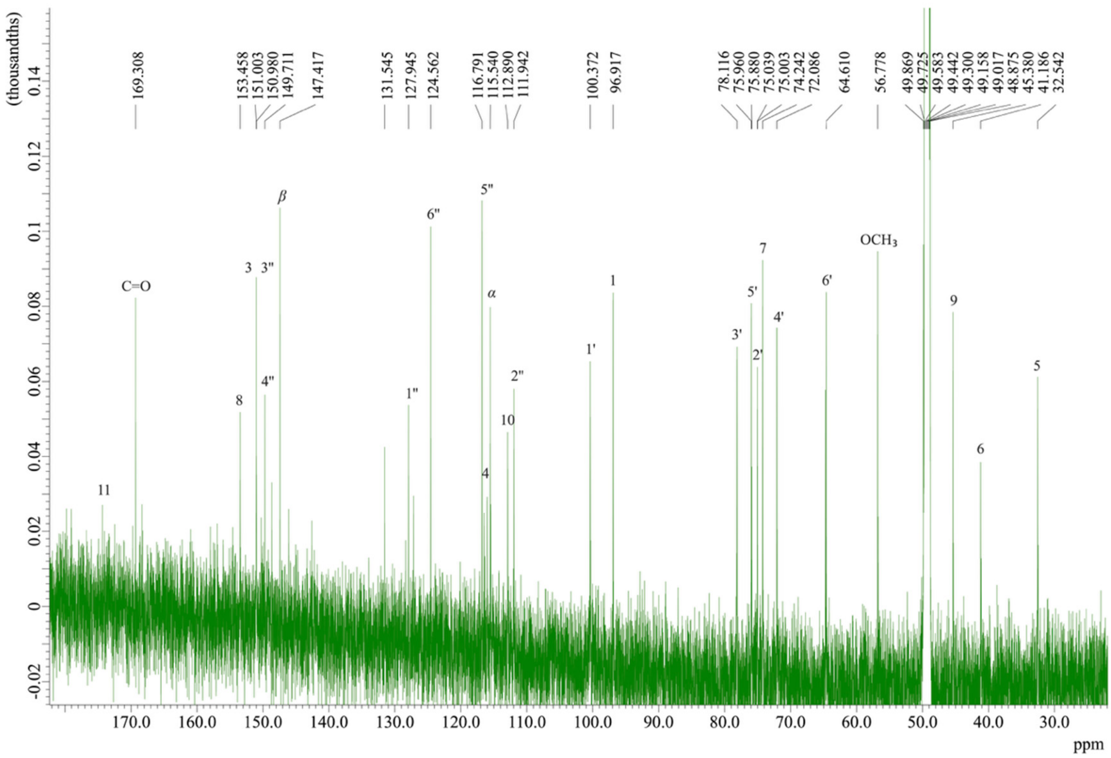

Figure S27: ^{13}C-NMR (150 MHz, CD_3OD) spectrum of **5** (feruloyl gardoside)

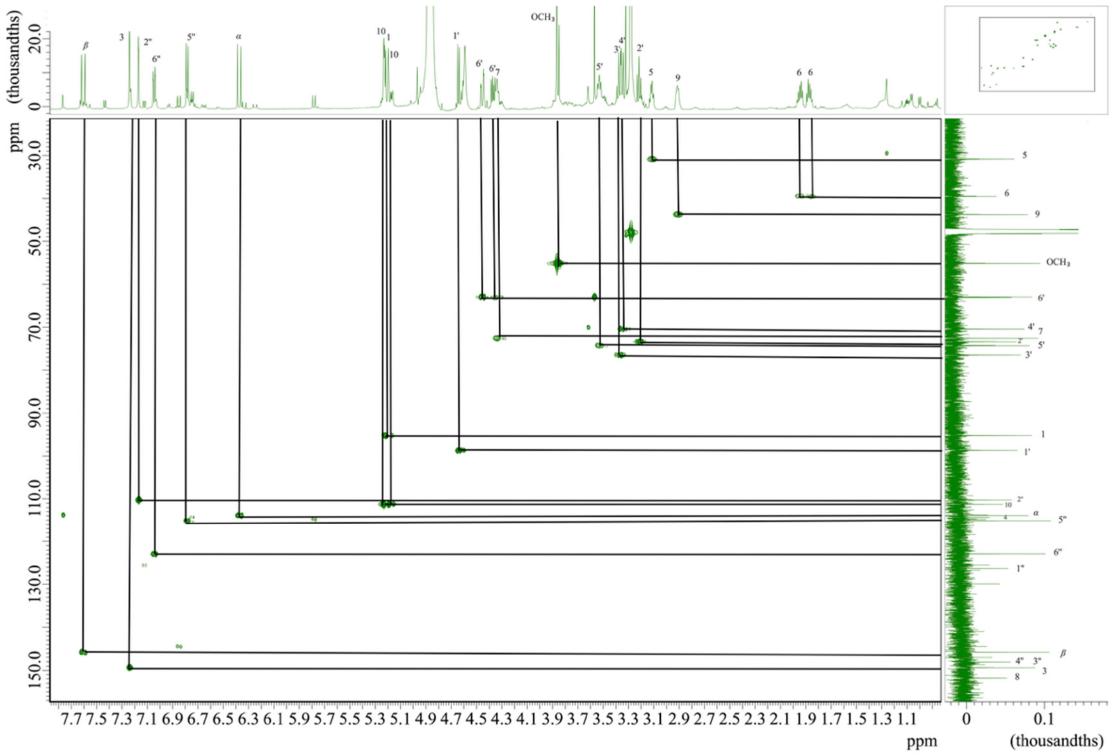

Figure S28: HMQC spectrum of **5** (feruloyl gardoside)

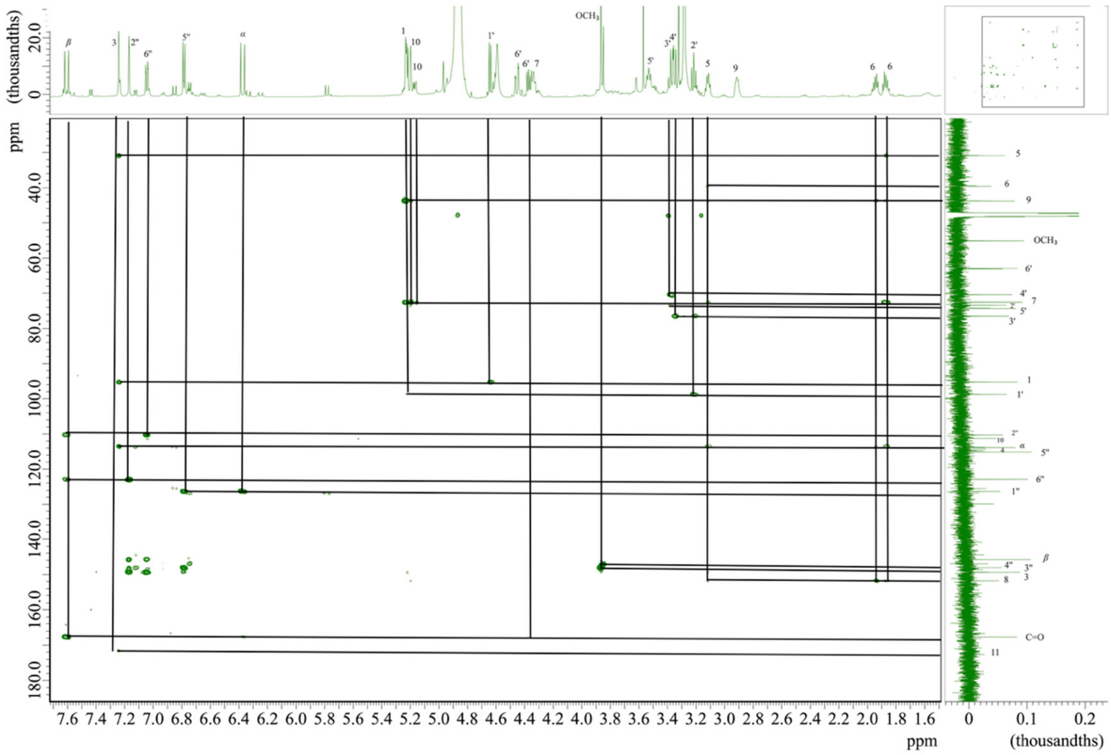

Figure S29: HMBC spectrum of **5** (feruloyl gardoside)

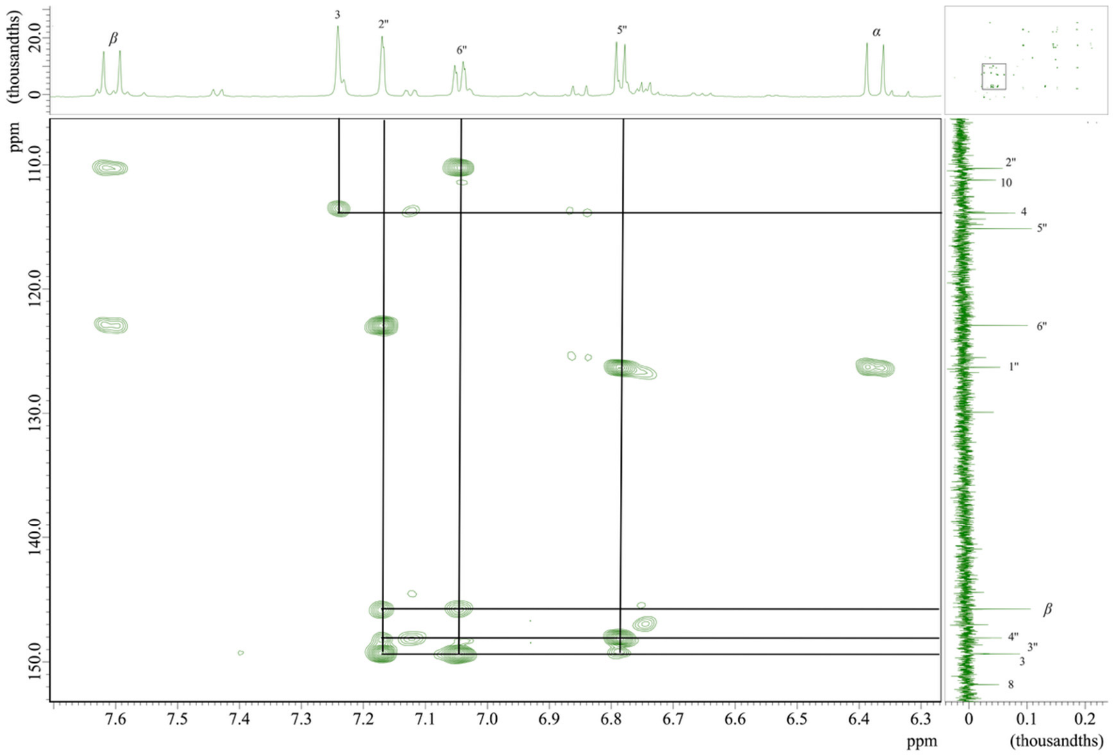

Figure S30: HMBC spectrum of **5** (feruloyl gardoside A) (From δ_C 107 ppm to δ_C 152 ppm)

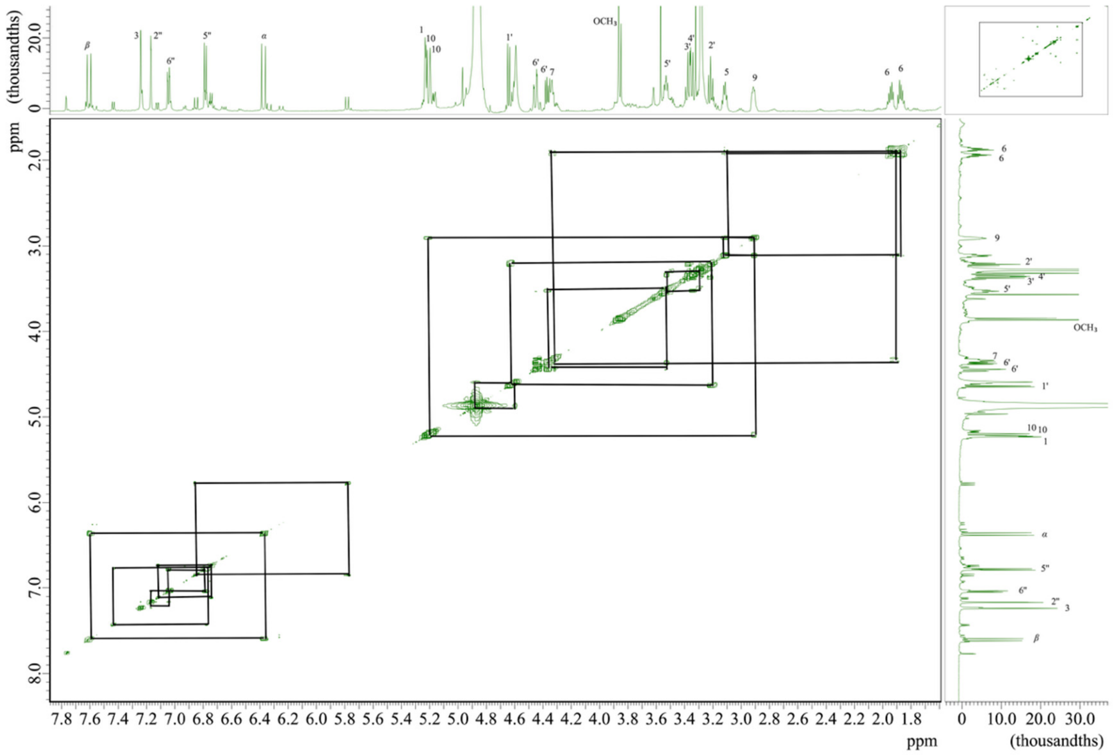

Figure S31: ^{1}H-^{1}H COSY spectrum of **5** (feruloyl gardoside)

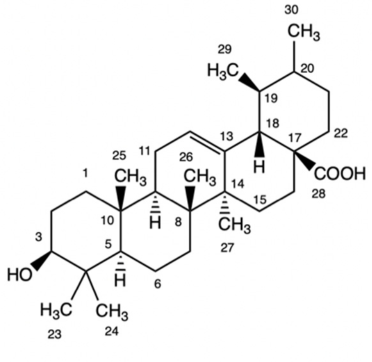

Figure S32: Chemical Structure of **6** (ursolic acid)

Ursolic acid: $C_{30}H_{48}O_3$ White Amorphous Powder ^{1}H NMR (600 MHz, C_5D_5N); δ_H 0.95 (1H, s, H-1a), 1.55 (1H, s, H-1b), 1.81 (2H, s, H-2), 3.45 (1H, brt, *J* = 6.0 Hz, H-3) 0.87 (1H, brs, H-5), 1.56 (1H, s, H-6a), 1.34 (1H, s, H-6b), 1.53 (1H, t *J* = 12.0 Hz, 7a), 1.36 (1H, t *J* = 12.0 Hz, 7b), 1.62 (1H, t, *J* = 9.6 Hz H-9), 1.93 (2H, s, H-11), 5.48 (1H, s, H-12), 1.27 (1H, s, H-15a), 2.31 (1H, t, *J* = 10.2 Hz, H-15b), 2.11 (1H, m, H-16a), 1.97 (1H, s, H-16b), 2.63 (1H, d, *J* = 11.4 Hz H-18), 1.44 (1H, s, H-19), 1.01 (1H, s, H-20), 1.37 (1H, s, H-21a), 1.46 (1H, brs, H-21b), 1.23 (1H, s, H-23), 1.01 (1H, s, H-24), 0.87 (1H, s, H-25), 1.04 (1H, s, H-26), 1.21 (1H, s, H-27), 0.99 (1H, d, *J* = 4.8 Hz, H-29), 0.94 (1H, d, *J* = 6.0 Hz H-30), ^{13}C NMR (150 MHz, C_5D_5N); δc 39.0 (C-1), 27.8 (C-2), 77.7 (C-3), 39.7 (C-4), 55.5 (C-5), 18.4 (C-6), 33.2 (C-7), 39.6 (C-8), 47.7 (C-9), 36.9 (C-10), 23.3 (C-11), 125.3 (C-12), 138.9 (C-13), 42.1 (C-14), 28.3 (C-15), 24.5 (C-16), 47.6 (C-17), 53.2 (C-18), 39.0 (C-19), 39.1 (C-20), 30.7 (C-21), 37.1 (C-22), 28.4 (C-23), 16.2 (C-24), 15.3 (C-25), 17.1 (C-26), 23.5 (C-27), 179.5 (C-28), 17.2 (C-29), 21.1 (C-30).; ESI-MS *m/z* 455.3 $[M-H]^-$. Data were compared with the literature [6].

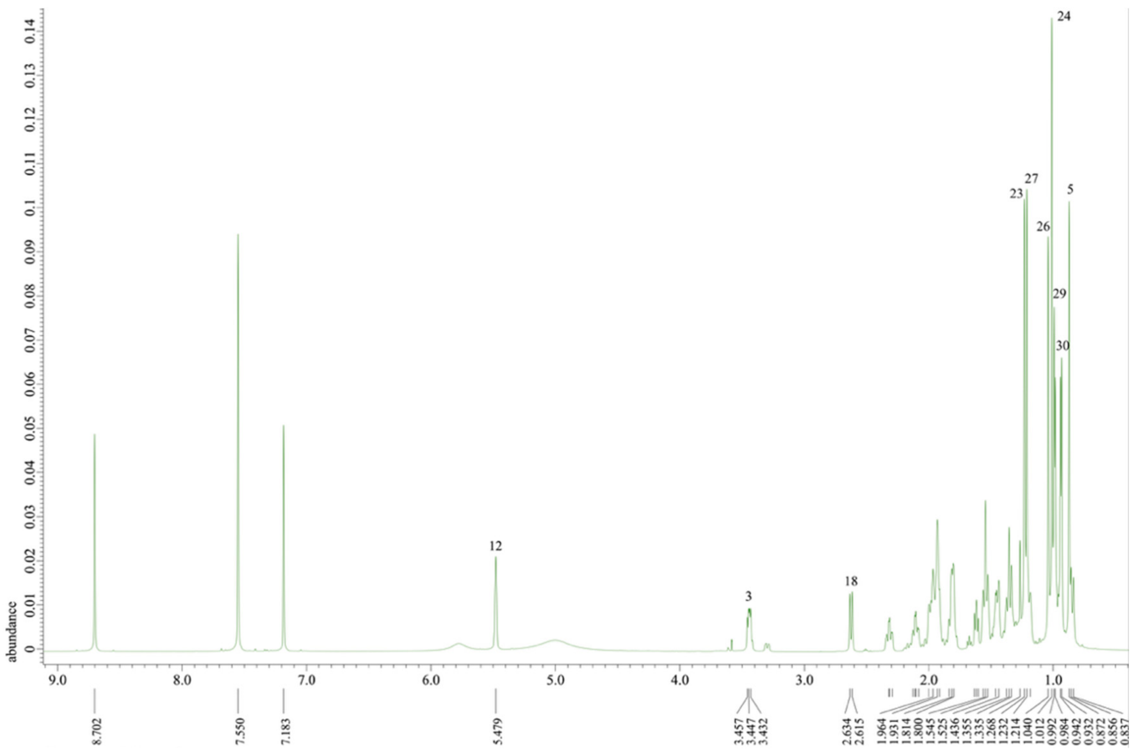

Figure 33: ^{1}H-NMR (600 MHz, C_5D_5N) spectrum of **6** (ursolic acid)

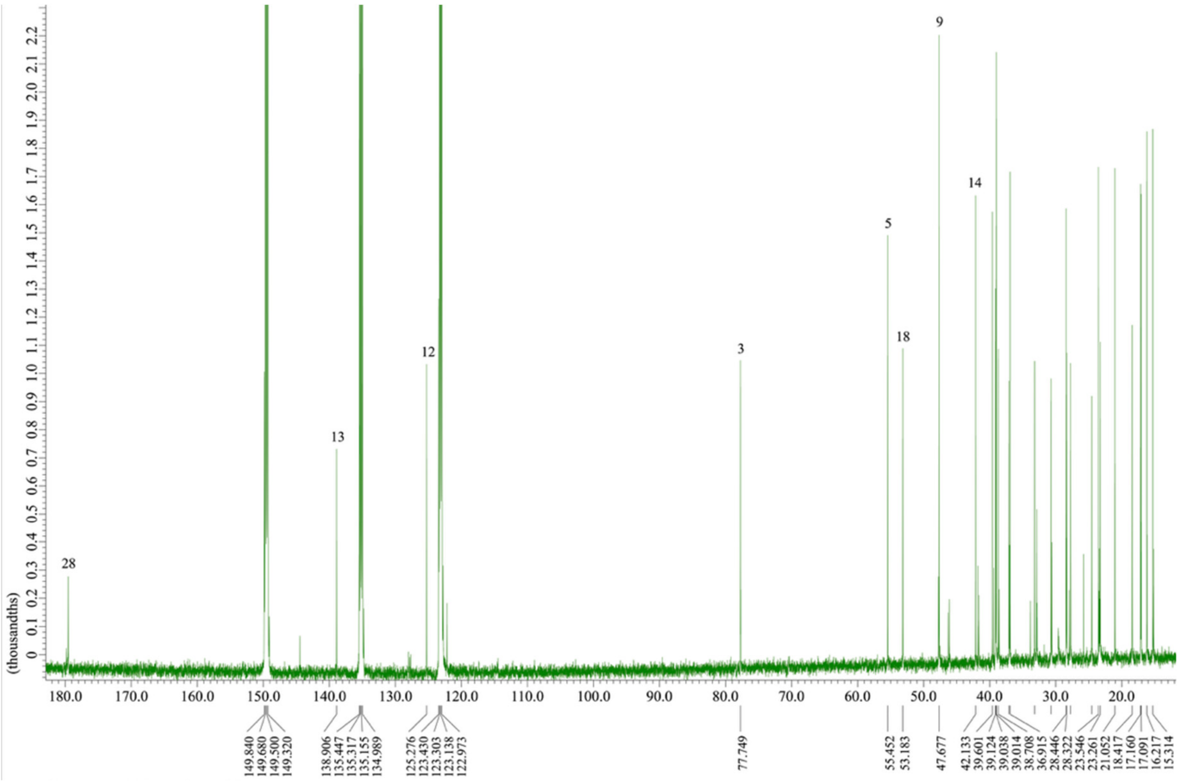

Figure S34: ^{13}C-NMR (150 MHz, C_5D_5N) spectrum of **6** (ursolic acid)

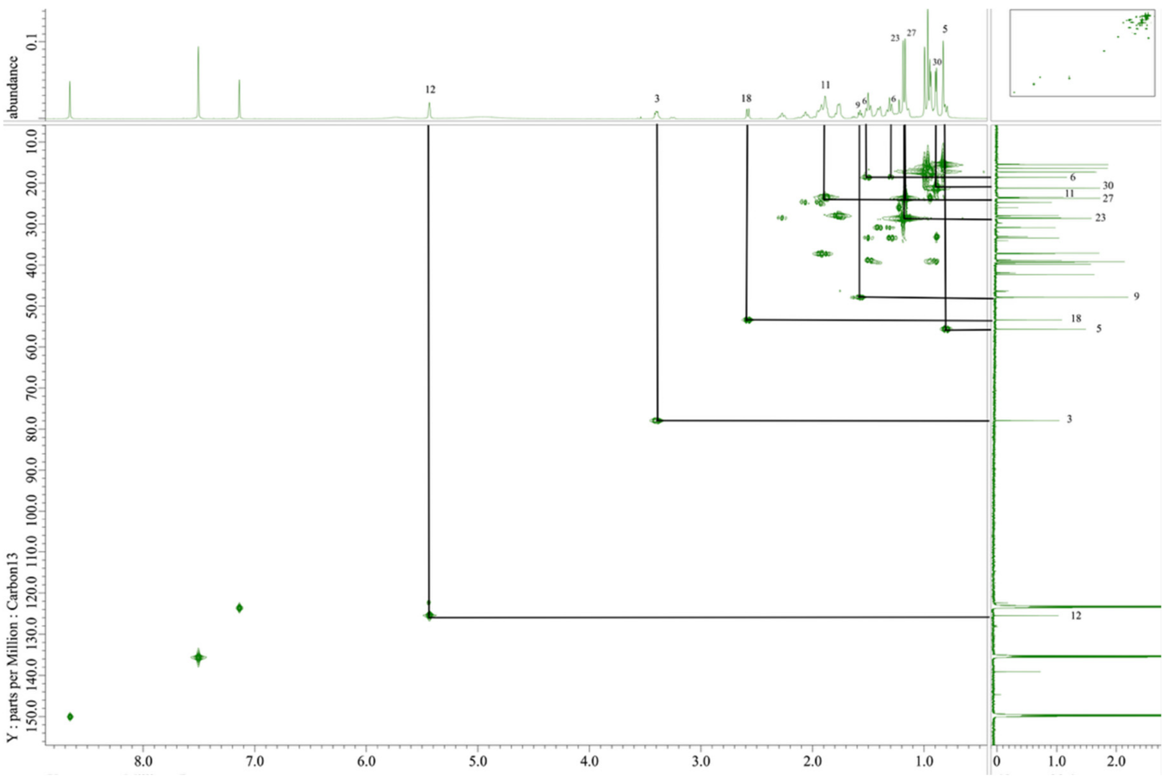

Figure S35: HMQC spectrum of **6** (ursolic acid)

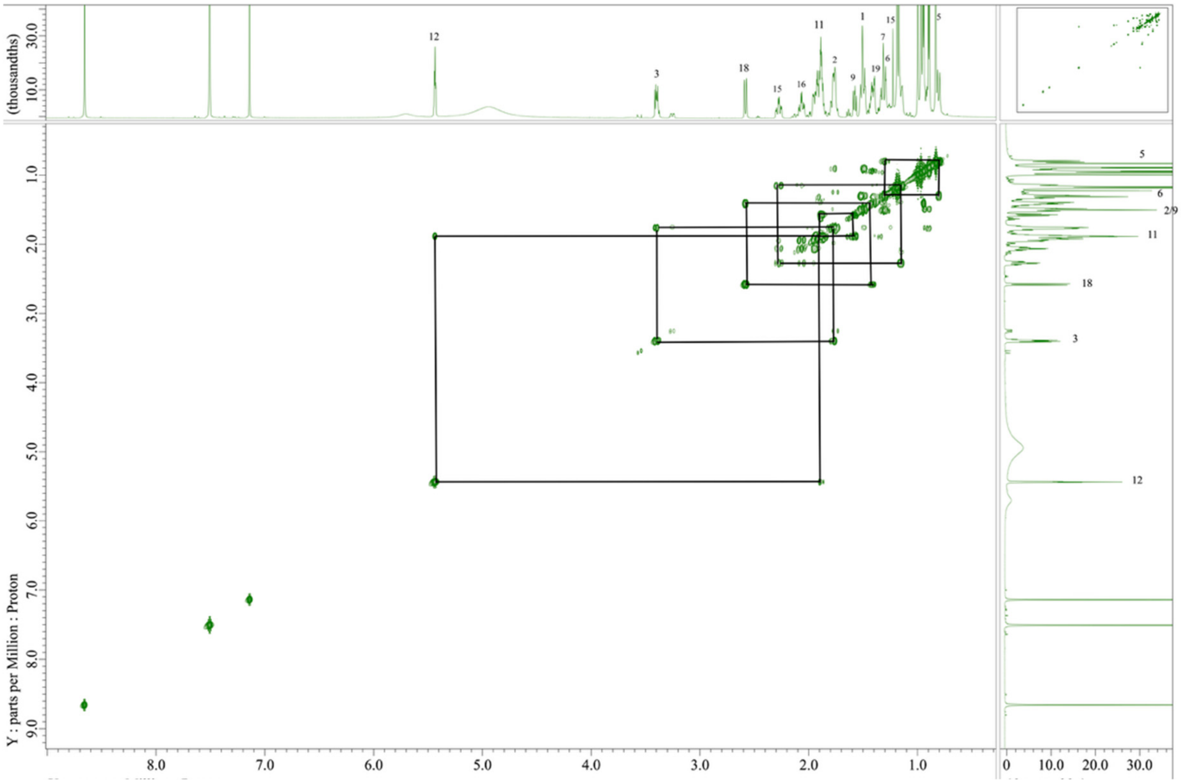

Figure S36: ^{1}H-^{1}H COSY spectrum of **6** (ursolic acid)

References

1. Andrzejewskagolec E, Ofterdingerdaegel S, Calis I, Swiatek L. Chemotaxonomic aspects of iridoids occurring in *Plantago* subg. Psyllium (Plantaginaceae). Plant Syst Evol. 1993; 185(1-2):85-89.
2. Pabst A, Barron D, Semon E, Schreier P. Two diastereomeric 3-oxo-α-ionol β-D-glucosides from raspberry fruit. Phytochemistry. 1992; 31(5):1649-1652.
3. Calıs I, Saracoglu I, Shizuka K, Sansei N. Phenylpropanoid glycosides isolated from *Rhynchocorys stricta* Scrophulariaceae. Turk J Med Sci. 1988; 12:234-238.
4. Genc Y, Harput US, Saracoglu I. Active compounds isolated from *Plantago subulata* L. via wound healing and antiinflammatory activity guided studies. J Ethnopharmacol. 2019; 241.
5. Chen X, Cao YG, Ren YJ, Liu YL, Fan XL, He C, Li XD, Ma XY, Zheng XK, Feng WS. Ionones and lignans from the fresh roots of Rehmannia glutinosa. Phytochemistry. 2022; 203:113423.
6. Seebacher W, Simic N, Weis R, Saf R, Kunert O. Complete assignments of ^{1}H and ^{13}C NMR resonances of oleanolic acid, 18α-oleanolic acid, ursolic acid and their 11-oxo derivatives. Magn Reson Chem. 2003; 41(8):636-638.
